# The impact of lutein-loaded poly(lactic-co-glycolic acid) nanoparticles following topical application: An *in vitro* and *in vivo* study

**DOI:** 10.1371/journal.pone.0306640

**Published:** 2024-08-01

**Authors:** Renee T. Carter, Sean Swetledge, Sara Navarro, Chin-C. Liu, Nikole Ineck, Andrew C. Lewin, Fabrizio Donnarumma, Ede Bodoki, Rhett W. Stout, Carlos Astete, Jangwook P. Jung, Cristina M. Sabliov

**Affiliations:** 1 Department of Veterinary Clinical Sciences, School of Veterinary Medicine, Louisiana State University, Baton Rouge, Louisiana, United States of America; 2 Department of Biological and Agricultural Engineering, Louisiana State University, Baton Rouge, Louisiana, United States of America; 3 Department of Entomology, Louisiana State University, Baton Rouge, Louisiana, United States of America; 4 Department of Chemistry, Louisiana State University, Baton Rouge, Louisiana, United States of America; 5 Department of Analytical Chemistry, “Iuliu Hatieganu” University of Medicine & Pharmacy, Cluj-Napoca, Romania; 6 Pathobiological Sciences, School of Veterinary Medicine, Louisiana State University, Baton Rouge, Louisiana, United States of America; Faculty of Sciences and Technology, State Islamic University of Sunan Kalijaga, INDONESIA

## Abstract

Antioxidant therapies are of interest in the prevention and management of ocular disorders such as cataracts. Although an active area of interest, topical therapy with antioxidants for the treatment of cataracts is complicated by multiple ocular anatomical barriers, product stability, and solubility. Entrapment and delivery of antioxidants with poly(lactic-*co*-glycolic acid) nanoparticles is a possible solution to these challenges, however, little is known regarding their effects in vitro or in vivo. Our first aim was to investigate the impact of blank and lutein loaded PLGA nanoparticles on viability and development of reactive oxygen species in lens epithelial cells in vitro. Photo-oxidative stress was induced by ultraviolet light exposure with cell viability and reactive oxygen species monitored. Next, an in vivo, selenite model was utilized to induce cataract formation in rodents. Eyes were treated topically with both free lutein and lutein loaded nanoparticles (LNP) at varying concentrations. Eyes were monitored for the development of anterior segment changes and cataract formation. The ability of nanodelivered lutein to reach the anterior segment of the eye was evaluated by liquid chromatography coupled to mass spectrometry of aqueous humor samples and liquid chromatography coupled to tandem mass spectrometry (targeted LC-MS/MS) of lenses. LNP had a minimal impact on the viability of lens epithelial cells during the short exposure timeframe (24 h) and at concentrations < 0.2 μg LNP/μl. A significant reduction in the development of reactive oxygen species was also noted. Animals treated with LNPs at an equivalent lutein concentration of 1,278 μg /mL showed the greatest reduction in cataract scores. Lutein delivery to the anterior segment was confirmed through evaluation of aqueous humor and lens sample evaluation. Topical treatment was not associated with the development of secondary keratitis or anterior uveitis when applied once daily for one week. LNPs may be an effective in the treatment of cataracts.

## Introduction

A cataract is characterized by opacification of the lens, which causes light passing through to scatter, resulting in impaired vision. A meta-analysis and review of global causes of blindness and severe visual impairment estimated that in adults 50 and older, 15.2 million people were affected by cataracts in 2020 resulting in blindness [1]. Cataracts can be classified as either acquired or congenital. Age-related cataracts are the most common type of acquired cataract in published reports [1, 2]. Currently, the only effective treatment for cataracts is surgical removal of a cataractous lens and replacement with an artificial lens. Access to vision-restoring surgical procedures is limited in some locations and is also associated with significant cost and risk of surgical complications [3]. Therefore, alternative therapies should be investigated to increase the availability of cataract treatment worldwide. Non-invasive treatments, such as topically applied eye drops, represent an ideal platform for the treatment of cataract as they allow patients to self-administer medications and avoid the risks associated with surgery.

Oxidative stress has been heavily implicated in the pathogenesis of cataract development [4, 5]. For oxidative stress to occur, increased levels of reactive oxygen species (ROS) are induced overwhelming natural antioxidant systems which are in place within the eye [6, 7]. Resulting damage to lens proteins, peroxidation of lens tissue and DNA damage culminate in cataract formation [2, 7]. As an increase in ROS in the aging eye have been reported, systemic antioxidant therapy has subsequently become a cornerstone in ocular health and a focus of cataract prevention strategies for many years [8–10]. Commonly utilized antioxidants include groups such as the carotenoids (lycopene, lutein, zeaxanthin, beta carotene) and vitamin E (alpha tocopherol). The effect of carotenoids on the incidence of age-related cataracts has been evaluated in multiple studies [8–11]. Increased dietary intake and plasma concentrations of lutein and zeaxanthin were associated with a reduction in risk for age-related nuclear cataract formation in the elderly [11].

Unfortunately, oral supplementation of antioxidant carotenoids, such as lutein, is limited by absorption in the gastrointestinal (GI) tract and first-pass metabolism limiting bioavailability to the lens [12]. Additionally, distribution to the lens is limited due to the lack of a direct blood supply with metabolic requirements met via the aqueous humor. Topical application of drugs to treat ocular disease is often utilized due to improved patient compliance, reduced systemic toxicity, non-invasiveness, and decreased risk of ocular complications [13]. However, most topically administered medications do not penetrate the cornea effectively and only approximately 5% of those will reach the intraocular tissues [14]. Furthermore, lutein is unstable and hydrophobic, and therefore it cannot be dissolved in conventional, saline based eye drop formulations for ocular delivery. Thus, a delivery vehicle is necessary for topical administration of lutein to the eye.

PLGA nanoparticles have been studied as a drug delivery platform given their biocompatibility, biodegradability, and ability to entrap and stabilize molecules of interest [14–16]. An improved outcome in cataract scores has been reported in a rodent selenite cataract model folloing topical treatment with lutein-loaded nanoparticles [12]. Although studies exist evaluating the benefit of systemic carotenoid (lutein and zeaxanthin) administration in relation to ocular health, little is known regarding the direct impact of free and nanodelivered lutein in vitro and in vivo.

Although the use of lutein-nanoparticles has been previously described in vivo [12], in this report, our goals were to expand our working knowledge regarding the in vitro effects of this delivery system and explore the ability to achieve desirable lutein concentrations within the eye following topical application. To accomplish our aims, PLGA nanoparticles were used to facilitate the delivery of lutein, a hydrophobic antioxidant, to human lens epithelial cell line (HLE) *in vitro* and to the eye of rodents in vivo. The impact of these particles on HLE cell viability, development of ROS in vitro was evaluated as well as the impact of lutein loaded nanoparticles (LNP) on photo-oxidative stress induced with UV light exposure. In vivo, eyes were monitored by slit lamp biomicroscopy and the ability of topically applied lutein delivered using LNP to penetrate the intraocular compartment and impact cataract formation was investigated.

## Materials and methods

### Materials

The following materials were obtained for nanoparticle thermosensitive gel synthesis from Sigma-Aldrich, Inc. (St. Louis, MO): 50:50 PLGA (38–54 kDa, cat. no. 719900), Tween-80 (cat. no. P1754), poloxamer 407 (P 407, cat. no. 16758) and 1.2% (w/w) poly(ethylene glycol) PEO 1105 (cat. no. 189456); lutein (Kemin Inc., Des Moines, IA); ethyl acetate (Fisher Scientific, LLC., Waltham, MA, cat.no. E196SK-4); D-(+)-Trehalose, dihydrate (Thermo Scientific, LLC., Waltham, MA; cat. no. 182551000). Materials utilized for nanoparticle suspension and in vitro work included Modified Eagle Medium (cat.no. 51200038), heat-inactivated fetal bovine serum (cat. no. 26140079), 1% penicillin/streptomycin (cat. no.15140122), and 0.25% trypsin (cat. no. 15050065) from Gibco, Thermo Fisher Scientific, Waltham, MA.

### Nanoparticle synthesis and characterization

PLGA nanoparticles loaded with lutein (LNPs) were synthesized by dissolving 50:50 PLGA and lutein in 8 mL ethyl acetate to form the organic phase. The compositions of the different LNP formulations used in this report are summarized in Table 1. Blank nanoparticles (BNP) were synthesized utilizing the same components without the addition of lutein. The aqueous phase consisted of Tween-80 dissolved in 80 mL ultrapure water (UPW, 18.2MΩ·cm) saturated with 8 mL ethyl acetate. The two phases were mixed to form a suspension under magnetic stirring for 10 min. The suspension was passed through a M-110P microfluidizer at 30,000 psi 3 times (Microfluidics Corp., Westwood, MA) as previously described [12]. Ethyl acetate was then evaporated using a rotavapor Buchi R-300 (Buchi Labortechnik AG, Flawil, Switzerland). D-(+)-Trehalose, dihydrate was added to the final suspension (3:1 w/w). The sample was placed at -80°C overnight then lyophilized using a freeze dryer (FreeZone 2.5L, Labconco Corporation, Kansas City, MO) for 48 h and then stored at -20°C until further use.

**Table 1:** Components for LNP synthesis.

Nanoparticle formulation	Polymer (mg)	Lutein (mg)	Surfactant (mg)	Trehalose (mg)	Final Lutein Conc. (μg/mL)	Nanoparticles concentration (mg/mL)
LNP, low	60	8	72	229	426	21.3
LNP, med	179	24	215	687	1278	63.9
LNP, high	300	40	360	1150	2130	106.5

The polymer, lutein, surfactant, and trehalose amounts used to make the nanoparticle formulations, the nanoparticle concentrations (low, medium, and high) and the equivalent final lutein concentration in these formulations are included.

Following synthesis, lyophilized nanoparticles were resuspended in UPW. Fresh and resuspended nanoparticle suspensions were analyzed via dynamic light scattering (DLS) using a Malvern Zetasizer (Malvern, United Kingdom) for size, polydispersity (PDI), and zeta potential. Nanoparticles were then prepared for in vitro and in vivo use. For in vitro studies, nanoparticles were resuspended MEM and used immediately. To increase corneal retention time for in vivo studies, all topical treatments were suspended in a thermosensitive gel that thickens at temperatures over 30–34°C prior to application [12]. To prepare the thermosensitive gel, a mixture of 15.6% (w/w) P 407 and PEO 1105 was utilized. Samples of either LNP or free lutein (for in vivo comparison purposes) were then added to the polymer components. UPW was added and samples were stirred at low temperature to avoid gel formation during preparation at the desired concentrations (Table 1) and stored at 4°C for the duration of the in vivo experiments (7 days). The final preparations did not contain any preservatives, but trehalose was added to the free lutein formulations to mimic the lyophilized formulations.

### Impact of lutein nanoparticles on HLE cells *in vitro*

The CellTiter-Glo 2.0^®^ (CTG) luciferase assay (Promega, Madison WI, USA; cat.no. G9241) was used to evaluate the viability of HLE cells following exposure to BNP or LNP synthesized as described in Nanoparticle Synthesis at the concentrations listed.

A human lens epithelial cell line transformed with adenovirus 12-SV40 virus hybrid (HLE B-3) was utilized (American Type Culture Collection, Manassas, VA, ATCC cat. no. CRL-11421). Lens epithelial cells (HLE) were cultured in phenol free MEM containing 10% heat-inactivated fetal bovine serum and 1% penicillin/streptomycin and cultured under standard environmental conditions. After reaching confluence, cells were trypsinized and 100μL (10,000 cells/well) of cell suspension was transferred to each well of a 96-well tissue culture plate.

After incubation of the 96 well plates at room temperature for 1 hour, each row of the 96 well plate was assigned to receive either control conditions or nanoparticle (NP, either BNP or LNP) at concentrations of 0.05 μg NP/μL, 0.25 μg NP/μL, or 0.50 μg NP/μL. Two types of control wells were included for each plate: 1) 100μL MEM media and no cells and 2) 100μL MEM media and 10,000 cells/well with no treatment. To prepare treatment, lyophilized BNP and LNP were resuspended in MEM to a concentration of 1 mg NP/mL (1 μg NP/μL). For all wells, a predetermined volume of media (5 μL, 25 μL, or 50 μL per well) was removed and a corresponding volume of either control (MEM media), BNP, or LNP were added to the respective well to maintain a total volume of 100 μL/well. All evaluations were performed in triplicate. Plates were incubated at 37°C and 5% CO_2_ for 24 h and then transferred to room temperature for 30 min immediately prior to performing the assay.

CTG reagent was added to each well, and cell lysis was induced using an orbital shaker at room temperature for 2 min. Following lysis, plates were incubated at room temperature for 10 min before luminescence was measured with a microplate reader (Synergy HTX, BioTek, Winooski, VT, USA). Cell viability was expressed as relative luminescence (RL). To calculate RL, the average of the blank control wells for each plate was subtracted from the reading obtained for each well. RL was then calculated as the percentage (fold change) compared to positive control well values for each plate. Each sample/concentration was evaluated in triplicate.

### Evaluation of reactive oxygen species (ROS) *in vitro*

The 2′,7′-dichlorofluorescin diacetate (DCFDA/H2DCFDA) cellular ROS assay kit (Abcam, Inc., Waltham, MA, cat. no. Ab113851) was utilized for in vitro ROS analysis. HLE cells were cultured and prepared as described above. A concentration of 10 μM DCFDA working solution was utilized and 50 μM *tert*-butyl hydroperoxide (TBHP) solution was freshly prepared as a positive control [17]. Cells were harvested and plated in 96-well microplates with a clear bottom and black sides at a concentration of 25,000 cells per well. Control wells and treatment assignment was similar to the methods described for CTG analysis. Plates were incubated as described above for 24 h; media was removed and 100 μL of DCFDA working solution was added to each well. The plates were then placed in a darkened incubator at 37°C for 45 minutes. DCFDA solution was then removed and 1× buffer was added. Results were immediately read on a microplate reader measuring fluorescence at excitation-emission spectrum of 485/535 nm. Results were calculated as described for CTG analysis. All sampling was performed in triplicate.

### Induction of HLE stress using ultraviolet B (UVB) *in vitro*

Ultraviolet light exposure is a known modifiable risk factor inducing photo-oxidative ocular stress [1]. To simulate photo-oxidative stress in vitro, HLE cells were cultured and treated with either control conditions or nanoparticle (NP, either BNP or LNP) at concentrations of 0.05 μg NP/μL, 0.25 μg NP/μL, or 0.5 μg NP/μL as previously described for 24 h. After 24 h cells were rinsed with blank MEM media. After 1 h, cells were exposed to UVB light (311 nm, 1.2 mW/cm^2^ × 250 s) for a total of 0.3 J/cm^2^. This power setting was chosen based on preliminary data which evaluated the viability of HLE cells following treatment ranging from 0.1 J to 0.6 J (311 nm, 1.2 mW/cm^2^ × 84 s-500 s) compared to controls (normal room lighting of equal duration). A 54.4% reduction in HLE cell viability for cells treated with 0.3 J/cm^2^ was identified, therefore, this setting was utilized for all remaining photo-oxidative stress experiments. Following UV exposure, plates were incubated for 24 h and CTG and ROS assays were performed as previously described. All sampling was performed in triplicate.

### Biodistribution and impact of lutein nanoparticles in vivo

To evaluate the ability of lutein delivered via a LNP platform to achieve detectable concentrations within the anterior segment of the eye, a pilot study was conducted utilizing adult Wistar rats (Envigo, Alice, Texas). All animals were housed in 12 h light/dark cycles with unrestricted access to food and water. Protocols were approved by the Institutional Animal Care and Use Committee of Louisiana State University (Animal Use Protocol 18–115, 22–042) and were conducted in accordance with the ARVO Statement for the Use of Animals in Ophthalmic and Vision Research. At the end of in vivo studies, animals were euthanized by placement in a CO_2_ chamber. Throughout the study, animals were monitored by laboratory veterinarians on site and treated in consultation with the veterinary staff or euthanized as above if pain or distress were noted.

Animals were randomly assigned (Random.org) to one of two experimental groups: a one-hour collection timepoint (4 animals) or a two-hour collection timepoint (4 animals). Eyes for each animal were also randomized (Random.org) to receive either test article or control. To prolong corneal contact of the applied test article, resuspended particles were added to a thermosensitive gel prepared as described in Nanoparticle Preparation. Control consisted of blank thermosensitive gel (without nanoparticle added). Eyes assigned to the treatment group were treated with 12 μL of LNP suspension with a lutein concentration of 1278 μg/mL within thermosensitive gel (8 eyes). The contralateral control eye received 12 μL of blank thermosensitive gel (8 eyes). Test article and control was administered using a calibrated micropipette by a single investigator.

Prior to application of test article or control, each animal was individually placed into an induction chamber connected to a calibrated isoflurane anesthesia unit. Oxygen flow rate was initiated at 1L/min and the flow of isoflurane began at 0.5% and was slowly increased until anesthesia was induced. After induction the animal was removed from the induction chamber and an anterior segment examination was performed by one expert examiner (RC) using slit lamp biomicroscopy (SL 17, Kowa, Tokyo, Japan). All animals were found to have a normal anterior segment examination prior to treatment. Following application of test article and control to both eyes, animals were allowed to recover in individual recovery cages during the incubation period.

Depending on the group assignment, animals were euthanized by placement into a CO_2_ gas chamber, either 60 min or 120 min following treatment. Immediately following euthanasia aqueous humor samples from each eye were collected by aqueous paracentesis method using a 30-gauge needle inserted into the anterior chamber. Aqueous humor samples were placed into individual Eppendorf tubes and immediately frozen on dry ice and stored at -80°C until analysis by liquid chromatography coupled to mass spectrometry (LC-MS). Whole globes were snap frozen and micro-dissected on dry ice for lens collection. Collected lenses were stored in individual Eppendorf tubes at -80°C until analysis by targeted LC-MS/MS separation.

LC-MS analyses of aqueous humor samples were conducted on an Agilent 1260 Infinity II quaternary liquid chromatograph coupled to an Agilent 6230 Electrospray Time-of-Flight mass spectrometer (Agilent, Santa Clara, CA). Samples were run with a capillary voltage of 4000V in positive mode. Nitrogen was used as drying gas delivered at 10 L/min at a temperature of 325°C, and the fragmentor voltage was set to 150 V. The mass range used was 100–3000 m/z. An Agilent Poroshell 120 EC-C18 column (2.7 mm ID, 150 mm length, 2.7 μm pores, end-capped) was used for chromatographic separation with a gradient program using a binary mixture of mobile phases at a fixed flow rate of 400 μL/min. Mobile phases composition was as follows: A = 0.1% formic acid in water, and B = acetonitrile (ACN). The gradient program was as follows: 0–5 min = 50% B, 5–30 min 90% B, 30–35 min 90% B, 35–45 min 50% B. A volume of 2μL was used for each injection and samples were run both in positive as well as in negative mode. Data were analyzed with MassHunter Workstation module Qualitative Analysis Navigator (Ver. B.08.00, Build 8.0.8208.0). Extracted ion chromatograms were generated using a value of + 568.428 m/z with a tolerance of 5 ppm, which was derived by comparison with a lutein standard (supplied from same stock utilized to produce free lutein and nanoparticle lutein formulations). Based on calibration curves performed using prepared lutein standards, the limit of quantification (LOQ) was 0.5 μg/mL (or 0.5 ppm).

Lens samples were prepared for analysis by resuspension in 100 μL of UPW with 0.1% formic acid. For extraction, 400 μL of n-hexane (Thermo Fisher Scientific, Waltham, MA, cat. no. LO9938-AK) was added and samples were vortexed, sonicated, and micro centrifuged to promote phase separation. The hexane phase was subsequently removed, quantified, and sample processing was repeated as listed above. Hexane fractions were pooled and dried under nitrogen for further analysis. After drying, samples were resuspended with 20 μL of methanol (Thermo Fisher Scientific, Waltham, MA, cat. no. 423955000) with 0.1% butylated hydroxytoluene (BHT, Sigma Aldrich, Inc., St. Louis, MO, cat. no. 1082708). Analysis utilized Targeted LC-MS/MS separation on a Waters Acquity Premiere UPLC system equipped with a Waters Acquity UPLC BEH C18 Column (130Å, 1.7 μm, 2.1 mm X 50 mm). The flow rate was set to 400 μL/min and the temperature was kept at 45°C. The analyte was separated with a gradient using two mobile phases: A = H_2_O, B = ACN, both containing 0.1% formic acid. The gradient program was as follow: 0–0.5 min 50% B, 0.5–3.5 min to 95%B, 3.5–4.5 min 95% B, 4.50–4.51 min to 50% B, 4.51–5.5 min 50% B. Samples were kept at 10°C within the autosampler and the injection volume was 1 μL.

Mass spectrometric measurements were conducted on a Waters Synapt XS Q-TOF mass spectrometer. The system was operated TOF MS/MS mode with the analyzer set to resolution mode. No targeted enhancement was employed, and the scan time was set to 2Hz. The scan range was set to 50–600 m/z with the fixed mass set to 568.428 m/z, which corresponds to the [M]·+ ion of lutein. The source was operated with a capillary voltage of 1V, the sampling cone was set to 25 V and the source offset was set to 30. The source temperature was set to 100°C, while the source desolvation temperature was set to 400°C. The cone gas was set to 20 L/h, the desolvation gas flow was 900 L/h and the nebulizer pressure was kept at 6.5 bar. Fragmentation experiments were performed in the transfer cell of the instrument with an energy of 14V, which produced two characteristic ions at 338.257 and 476.3645 m/z, which were used to create extracted ion chromatograms for signal integration.

Targeted analysis data was analyzed with MassLynx V.4.2 (SCN1028). Extracted ion chromatograms were generated with a mass windows of 0.02 m/z and smoothed with a Savitzky Golay algorithm (3 scan window, 1 iteration). Peak detection and integration were performed manually. Peaks with S/N < 10 were considered below the limit of quantification (BLOQ). Control and treated lenses at one hour were analyzed individually; due to low peaks obtained using single lenses, samples obtained at 2 hours (control 2-hour and treated 2-hour) were pooled for further analysis. Lens sample analytes were compared to prepared lutein calibration curves. A polynomial trendline was generated (correlation coefficient r = 0.9847) and utilized to calculate the estimated lutein concentration of lenses by measuring the area under the curve for two characteristic ions at 338.257 and 476.3645 m/z.

### Treatment effect on selenite-induced cataract formation in vivo

**Fig 1:**
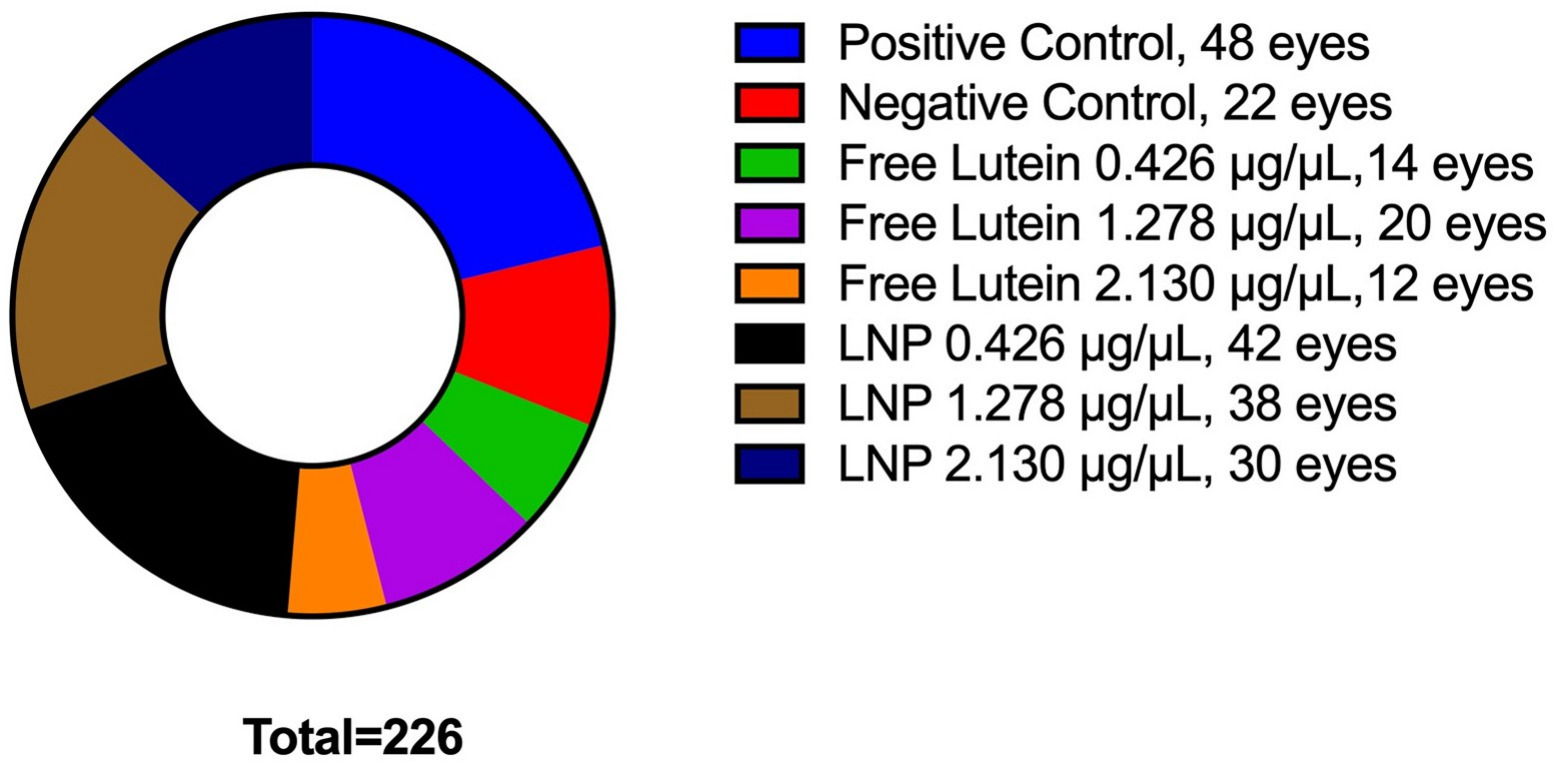
Allocation of animals by group. Pie chart depicting the allocation of animals by litter to randomly assigned treatment or control groups.

Wistar rat pups (Envigo, Alice, Texas) were obtained and were used to assess the ability of LNP to reduce cataract formation in vivo utilizing a selenite model [18]. Pups were housed and cared for as previously described and randomly assigned by litter into either one of two control groups or one of six treatment groups listed in Table 2. The six treatment groups included three LNP delivery formulations and three equivalent free lutein formulations added to a thermosensitive gel prepared as previously described. A total of 113 rats (226 eyes) were available for study. The allocation for these animals is depicted in Fig 1; animals were randomly allocated to group by litter, therefore, litter size impacted distribution of animals between groups.

**Table 2:** Design for experimental control and treatment groups.

Study Group	Intraperitoneal Treatment
Positive control	Intraperitoneal Selenite Injection
Negative control	Intraperitoneal Saline Injection
Free Lutein 426 μg/mL	Intraperitoneal Selenite Injection
Free Lutein, 1278 μg/mL	Intraperitoneal Selenite Injection
Free Lutein, 2130 μg/mL	Intraperitoneal Selenite Injection
LNP, 21.3 mg/mL (equivalent to 426 μg/mL lutein)	Intraperitoneal Selenite Injection
LNP, 63.9 mg/mL (equivalent to 1278 μg/mL lutein)	Intraperitoneal Selenite Injection
LNP, 106.5 mg/mL (equivalent to 2130 μg/mL lutein)	Intraperitoneal Selenite Injection

Summary of design for experimental control and treatment groups where LNP denoted lutein loaded nanoparticles.

At 13-day post-partum, rat pups were weighed and received an intraperitoneal injection of sodium selenite dissolved in 1× phosphate-buffered saline (PBS) at 30 μmol/kg body weight (BW) to induce cataract formation. Weights across litters of animals were normally distributed (as evaluated by Shapiro-Wilk, skewness, kurtosis, and q-q plots) and ranged from 17.9 to 34.6 grams (mean 22.4 grams, 95% CI [21.89, 22.92]). Animals assigned to the negative control group were given an intraperitoneal injection with an equal volume of 1× phosphate-buffered saline (PBS) without selenite. Positive controls received selenite injections but received no topical treatments, Table 2.

Topical treatments were instituted on day 14 post-partum, 24 h after selenite or PBS intraperitoneal injection. For animals in the treatment groups, both eyes were treated with one drop (12 μL) of the lutein-containing formulations, for seven consecutive days at the same time each day by the same investigator. Test article was administered using the same calibrated micropipette.

The eyelids of some animals were still sealed on the first day of topical application, necessitating that the eyelids be gently opened manually prior to application. After treatment application, animals were kept separated from their dam for 30 min to reduce cleaning of the application site, thus increasing retention time on cornea.

After seven days of treatment, a boarded veterinary ophthalmologist (RC), blinded to the treatment groups, performed an anterior segment examination. Pharmacological mydriasis was induced prior to examination using 1% tropicamide (Alcon Laboratories, Inc., Fort Worth, Texas, USA). Each eye was evaluated for the presence of anterior segment abnormalities by slit lamp biomicroscopy (SL 17, Kowa, Tokyo, Japan). Each lens was evaluated for the presence of opacity, location of opacity (cortical, nuclear) and percentage of lens involvement (from 0–100%). A cataract scoring system was developed based on previous reports evaluating cataracts in rodent experimental models [19, 20]. Using the parameters described in Table 3, cataracts were graded and assigned to a stage from 0–6.

**Table 3:** Cataract grading.

Stage 0	Normal transparent lens
Stage 1	Initial hazy nuclear opacity or incipient cortical opacity (one location)
Stage 2	Initial hazy nuclear opacity and incipient cortical opacity (two or more locations)
Stage 3	Dense partial nuclear cataract and incipient cortical opacity (< 25%)
Stage 4	Dense partial nuclear cataract and incipient cortical opacity (≥ 25%, <75%)
Stage 5	Dense partial nuclear cataract and incomplete cortical opacity (≥ 75%, <100%)
Stage 6	Complete cataract, nuclear and cortical (100%)

Cataract stages grading parameters as determined by slit lamp biomicroscopy.

### Statistical analysis

Data analyses were performed using JMP Pro 16.2.0 (SAS Institute, Cary, NC). Graphs were generated using Prism 9 for Windows, Version 9.5.0 (GraphPad software, LLC, San Diego, CA). For *in vitro* experiments, the associations of ROS and CTG with different treatments were evaluated via ANOVA with Dunnett’s post hoc pairwise comparisons to control samples. Assumptions of the parametric models, normality of residuals and homoscedasticity, were assessed by examining standardized residual and quantile plots. For *in vivo* studies, cataract scores were evaluated using a Kruskal-Wallis with Dunn’s post hoc comparison to positive control. Data are presented as mean SD. Significance was set at p < 0.05.

## Results

LNPs were found to have an average diameter of 201± 3 nm; BNPs were of a similar average diameter of 260 ± 6 nm (Table 4), at the lower size range of particle measuring 100 nm to 3000 nm that have been reported for ocular delivery of therapeutic compounds [21]. A PDI of 0.166 ± 0.009 was obtained for LNPs and of 0.160 ± 0.004 for BNPs. These low PDI values indicate a narrow size and uniform colloidal distribution [22]. A negative zeta potential was obtained for both formulations (LNP -36.6 ± 8.4 mV, BNP -33.0 ± 0.5mV) and is ideal to ensure a uniform and stable colloidal dispersion of the nanoparticle formulations. Additionally, negatively charged PLGA nanoparticles reportedly do not induce significant cytotoxic effects in vitro [15].

**Table 4:** Nanoparticle characterization.

Nanoparticle formulation	Average diameter (nm)	PDI	ζ-potential (mV)
LNP	201 ± 3	0.166 ± 0.009	-36.6 ± 8.4
BNP	260 ± 6	0.160 ± 0.004	-33.0 ± 0.5

Nanoparticle diameter, Polydispersity Index (PDI) and Zeta potential (ζ-potential) were determined based on Dynamic Light Scattering (DLS) measurement.

### Nanoparticle impact on HLE cell viability in vitro

**Fig 2:**
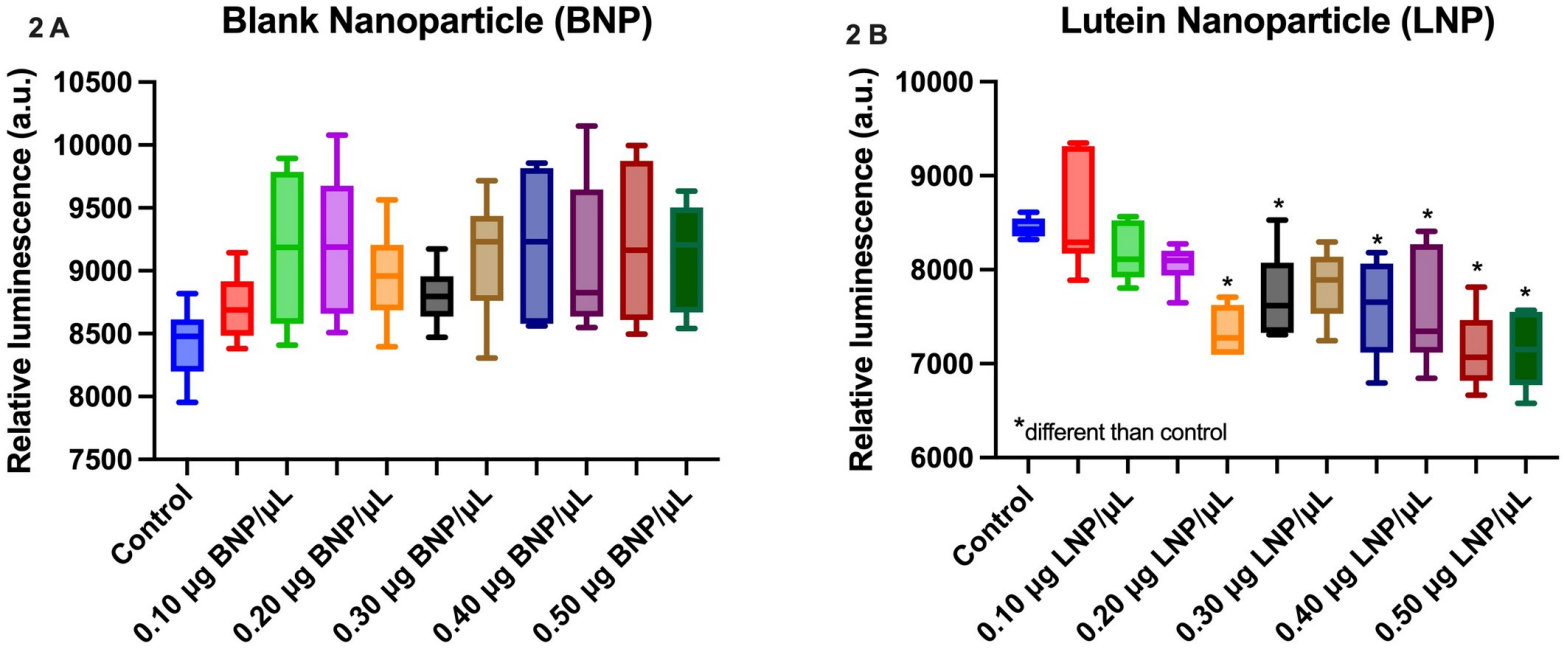
Impact of nanoparticles on HLE cells in vitro. Box and Whisker plots representing the impact of BNP (Fig 2A) and LNP (Fig 2B) on HLE cells at concentrations of 0.05–0.50 μg of respective NP/μL. Cell viability was calculated by comparing relative luminescence (arbitrary units, a.u.) to control. A reduction in HLE cell viability was identified during the exposure timeframe (24 h) and statistically significant at concentrations of 0.20 μg LNP/μL and higher for LNP compared to control (0.2 μg LNP/μL, p = 0.0003; 0.25 μg LNP/μL, p = 0.0311; 0.35 μg LNP/μL, p = 0.0068; 0.40 μg LNP/μL, p = 0.0050; 0.45 μg LNP/μL, p<0.001; 0.50 μg LNP/μL, p<0.001). No difference was observed for BNP (p > 0.05). p<0.05*.

BNPs and LNPs were evaluated in vitro utilizing MEM media as a diluent and control. Concentrations of NP from 0.05 μg/μL to 0.50 μg/μL (for both BNP and LNP) were evaluated. A change in cell viability following exposure to BNP and LNP is expressed as relative luminescence (RL) compared to control, Fig 2A and 2B. A minimal impact on cell viability was identified during the short exposure timeframe (24 hours) for BNP formulations. However, a significant reduction in cell viability was noted at concentrations of 0.20 μg LNP/μL (p = 0.0003) to 0.50 μg LNP/μL (p = 0.0001) of LNP when compared to control and to BNP tested concentrations (one way ANOVA with Dunnett’s post hoc pairwise comparisons).

**Fig 3:**
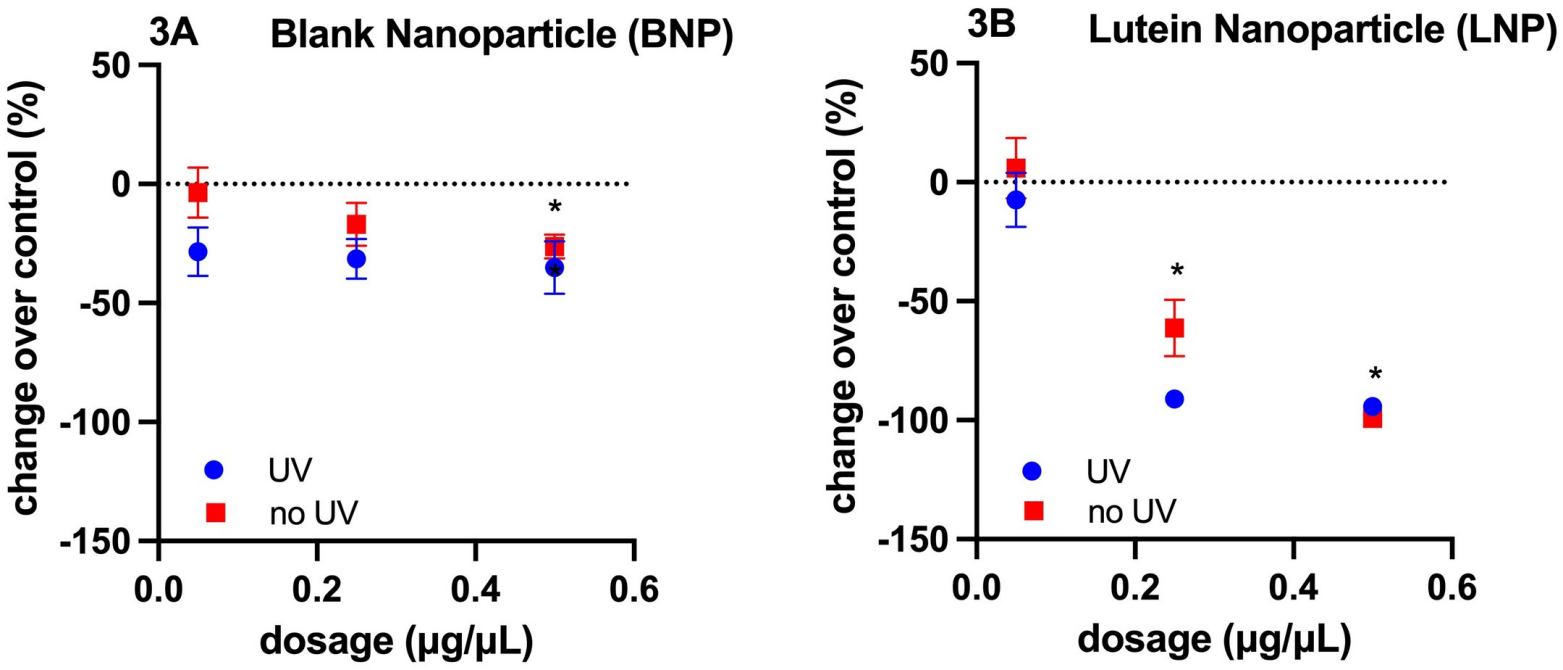
HLE cells viability when exposed to UV in the presence of nanoparticles. HLE cells in the presence of 0.50 μg BNP/μl, 0.25 μg LNP/μL, and 0.50 μg LNP/μL, had a significant reduction in viability compared to cell control (p = 0.0119, <0.0001, <0.0001, respectively). To calculate change over control (% change), average values for blank control wells were subtracted from the reading obtained for each tested well and compared to positive control values for each plate. p< 0.05*.

HLE cells were then exposed to UV light to simulate photo-oxidative stress. UV light exposure had a significant effect (p = 0.0010) on cell viability when compared to controls (with UV -50.1 ± 3.0%, and without UV -35.8 ± 3.0%). HLE cells in the presence 0.50 μg BNP/μL, 0.25 μg LNP/μL and 0.50 μg LNP/μL, had a significant reduction in viability compared to cell control (p = 0.0119, <0.0001, <0.0001, respectively, utilizing an ANOVA with Dunnett’s post hoc pairwise comparisons) Fig 3.

**Fig 4:**
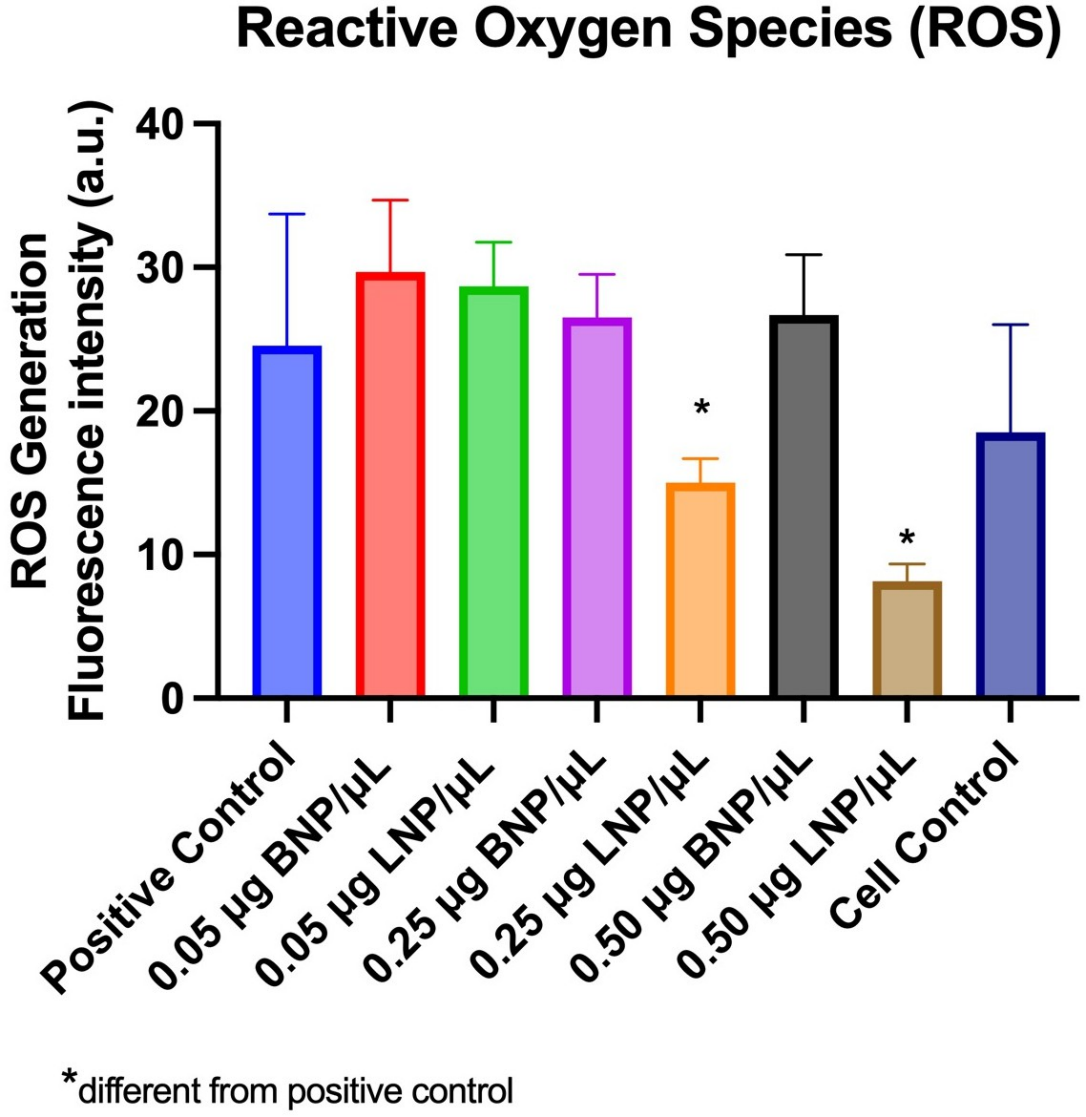
Reactive oxygen species (ROS) concentrations following exposure to nanoparticles. Following exposure to BNP and LNP at concentrations of 0.05 μg NP/μL, 0.25 μg NP/μL and 0.50 μg NP/μL, a change in fluorescence (arbitrary units, a.u.) was used to quantify the level of ROS and calculated by subtracting measurements from blank controls (0.25 μg LNP/μL mean 22.92, ± SD 3.08, p = 0.0321; 0.50 μg LNP/μL mean 2.42, SD ±1.17, p = 0.0001). p*< 0.05.

**Fig 5:**
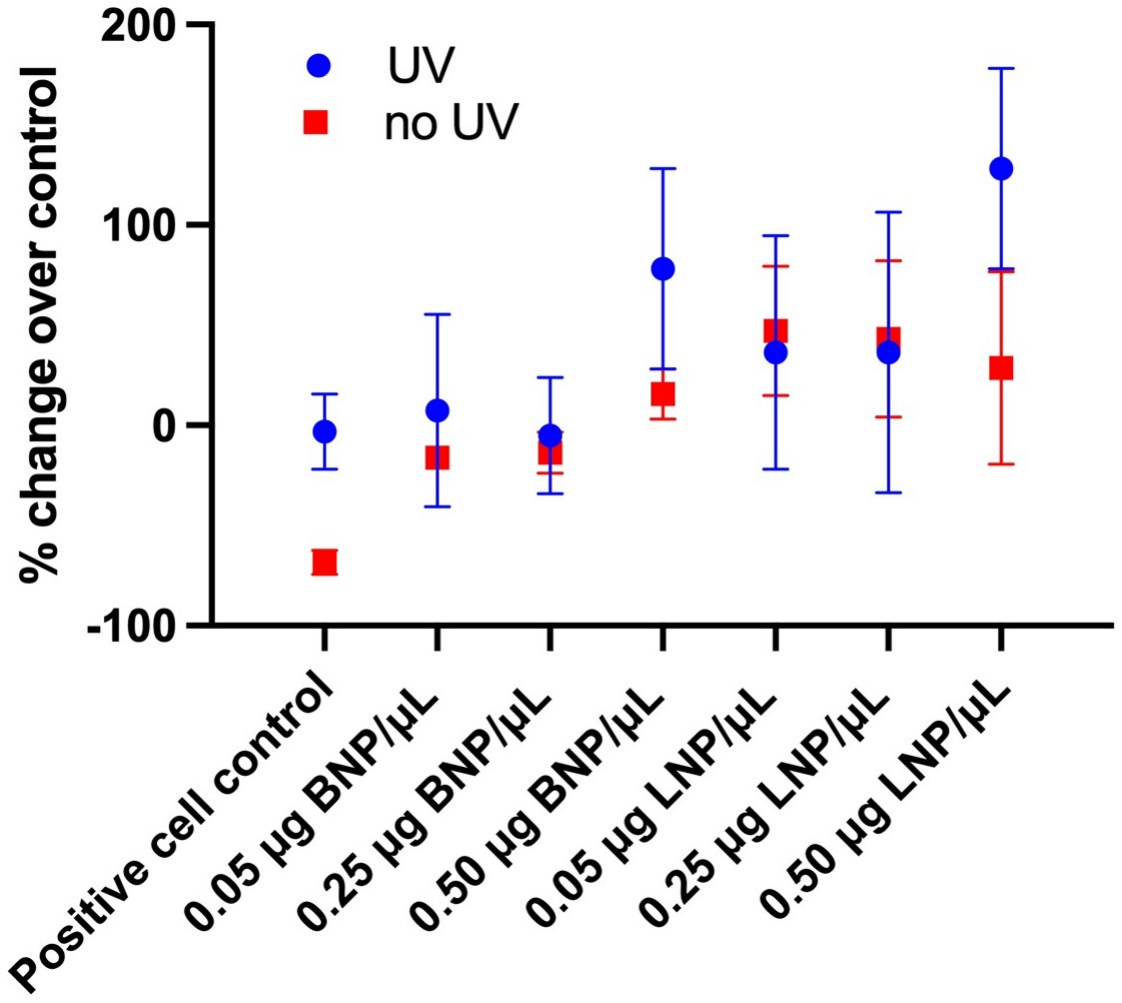
Reactive oxygen species (ROS) concentrations following exposure to UV in the presence of nanoparticles. Following UV light exposure, levels of ROS increased in all tested groups (BNP and LNP) compared to controls and approached significance (p = 0.0828). Results were calculated as a percent change over control (with UV 43.8 ± 22.2%, without UV -12.1 ± 22.2%). No significant difference with either BNP or LNP was identified.

Levels of ROS increased with exposure to both BNP and LNP nanoparticles *in vitro*. However, this increase appeared to be a dose dependent effect, as a significant impact on ROS, independent of nanoparticle matrix, was identified. At concentrations exceeding 0.25 μg LNP/μL, a reduction in ROS was identified compared to BNP and cell control, Fig 4, one-way ANOVA with Dunnett’s *post hoc* pairwise comparisons to control samples. Following UV light exposure, levels of ROS increased in all tested groups (BNP and LNP) compared to controls and approached significance (p = 0.0828) Fig 5. Results were calculated as a percent change over control and there was no significant difference in BNP or LNP in this effect (one way ANOVA with Dunnett’s post hoc pairwise comparisons to control samples).

### Biodistribution and impact of lutein nanoparticles in vivo

Aqueous: An average of 10 μL of aqueous was collected from each control and treated eye. Due to the limited quantity of material available for study, aqueous was pooled by group (control 1 h, control 2 h, treatment 1, treatment 2 h) for analysis by LC-MS and compared to freshly prepared lutein standards. Blanks were run between each sample during analysis.

Extracted ion chromatograms of lutein from all the samples and the standard, as well as the spectra of the peaks were identified. While lutein was detected in both the one hour and two hour treated aqueous samples, the signal was too low to quantitate for statistical comparison (< 0.5 μg/mL or 0.5 ppm, S1–S2 Figs). Lutein was not detected in pooled aqueous for either the one-hour or two-hour control samples analyzed.

Lenses: Whole lenses collected from each control and treated eye were analyzed. Initially, a one-hour control and a one-hour treated lens sample were evaluated individually. However, due to the limited quantity of material available for study, lenses were later pooled by group (Control 2 h, Treatment 2 h) for targeted LC-MS/MS analysis and compared to freshly prepared lutein standards. Extracted ion chromatograms of lutein from all the samples, the standard, as well as the spectra of the peaks were identified (S3–S7 Figs). While lutein was detected in all lens samples, values were considered below the limit of quantification (LOQ) and therefore too low to quantitate for statistical comparison. Calculated values of lutein in attograms (ag) were highest in lenses collected one-hour post treatment application and were reduced at the 2h time point (Table 5).

**Table 5:** Estimated attograms (ag) of lutein from lens samples.

Lens sample	Sample AUC	Estimated sample lutein, ag
**Control, 1 hour**	41	1.3
**Treatment, 1 hour**	533	16.9
**Control, 2 hour (pooled)**	71	2.3
**Treatment, 2 hour (pooled)**	170	5.5

Estimated attograms (ag) of lutein for lens samples based on the area under the curve (AUC) of two characteristic ions for lutein (338.257 and 476.3645 m/z). AUC was highest within 1 h treated lens samples.

Together these data evaluating biodistribution support that lutein, delivered via an LNP platform, penetrates the ocular surface and reaches detectable levels within the aqueous and lens of rats following a single topical application. Clearance from the anterior segment is rapid as concentrations reduce within 2 hours of treatment.

### Treatment effect on selenite-induced cataract formation in vivo

**Fig 6:**
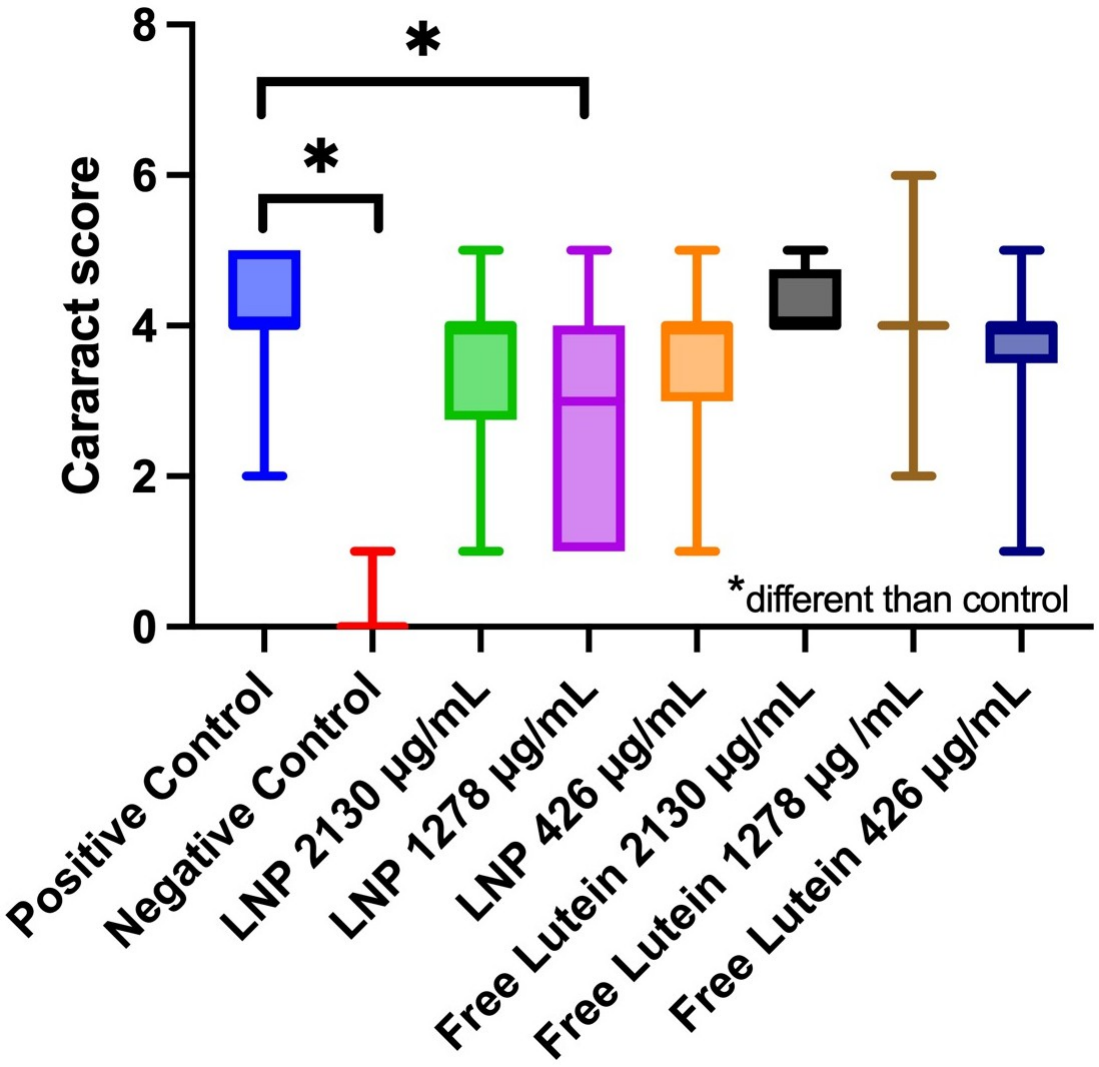
Cataract score per treatment group. Free lutein and LNP were tested at equivalent lutein concentrations of 426 μg/mL, 1278 μg/mL, and 2130 μg/mL lutein. Groups treated with 1278 μg/mL had significantly lower cataract scores (median 3, IQR [4–1]) than the positive control group (p = 0.0025). *p < 0.05.

Examination of all animals by slit lamp biomicroscopy showed no evidence of keratitis or anterior uveitis in any eyes of the treatment or control groups at the end of the experimental period. The effect of eye (right vs. left) was evaluated and found not to be a significant factor for cataract formation. Average cataract scores for animals treated with PLGA nanoparticles and free lutein at different lutein doses are represented in Fig 6. Groups treated with 1278 μg/mL nano delivered lutein had significantly lower cataract scores than the positive control group which received selenite but no topical treatment (p = 0.0025, Kruskal-Wallis with Dunn’s comparison to positive control). The 1278 μg/mL nano delivered lutein group had the lowest average cataract score (median 3, IQR [4–1]) among treatment groups but was not significantly different from the 2130 μg/mL nano delivered lutein group (median 4, IQR [4–1]. There was no significant difference in cataract score for any of the free lutein formulations.

## Discussion

Oxidative stress represents an imbalance in pro-oxidants and antioxidants due to either an increase in the level of ROS or a decline in antioxidant protection systems [23]. Oxidative stress is heavily implicated in the pathogenesis of age-related cataracts [5, 24]. Compression and hardening of the lens with age impairs the circulation of water and antioxidants through the lens which are needed for homeostasis, resulting in post-translational modification of α and β crystallins, including misfolding, oxidation, and truncation [4, 5]. A cataract subsequently results from increasing levels of oxidation products, decreased innate antioxidant components, and protein modification [4, 24–26]. Therefore, antioxidant therapies which can reduce oxidation and maintain homeostasis of lens proteins can potentially reduce cataract progression.

Several topical formulations have been tested for their ability to reverse cataract formation, including chaperone proteins and sterols with surfactant properties [27, 28]. These strategies for reversing cataracts are generally directed at returning misfolded crystallins to their native conformations and solubilizing them. While this strategy may be effective for congenital cataracts caused by mutated crystallin proteins, it would likely have little to no positive effect for senile cataracts where crystallins are chemically altered via oxidation and truncation [24].

Natural cataract prevention strategies using antioxidants, such as lutein, are of interest due to their potential to reduce photo-oxidative stress and consequently reduce post-translational protein modification. These naturally occurring carotenoid pigments are widely available as supplements designed to support ocular health [29]. Lutein is normally found in the lens epithelium and lens cortex, but the content often declines with age and a reduction in dietary intake [30–32]. While the function of lutein in the eye is not fully characterized, it is theorized that during oxidative stress, the OH functional group of lutein is able to react with free radicals thereby protecting membrane phospholipids from oxidative injury [32]. Due to the presence of extensive conjugated bonds, lutein also absorbs high energy blue light, thus reducing photo-oxidative stress, contributing to its protective role in the eye [33–35]. The effectiveness of lutein oral supplementation in ocular health is limited by both absorption in the GI tract and distribution to the eye [12, 36, 37]. Furthermore, lutein is unstable and hydrophobic, and therefore it cannot be dissolved in conventional, saline based eye drop formulations for ocular delivery. Thus, a delivery vehicle is necessary for topical administration of lutein to the eye. Specifically, copolymers such as PLGA are more advantageous for drug delivery systems when compared to homopolymers as they are reportedly biodegradable, biocompatible, tunable, and can be functionalized [38].

In phase one of this study, we investigated the impact of LNP on HLE cells, with and without photo-oxidative stress (UV exposure) (Figs 2–5). Cell type has been reported as an important factor when assessing nanoparticle toxicity in vitro [15], however, the impact of exposure to LNP on HLE cells has not been previously evaluated to the authors’ knowledge. LNP reduced the viability of HLE cells without UV exposure, however, this effect was concentration dependent and only occurred at higher concentrations (between 0.20 μg LNP /μL to 0.50 μg LNP/μL, Fig 2). A similar finding was not identified for BNP, therefore the impact on the extent of HLE cell viability was not due to the PLGA or surfactant used in nanoparticle production.

There are conflicting reports on the impact of the concentration of free lutein on cell viability [23, 39]. Incubation of HLE cells with 0.1 to 10 μM of lutein was found to have no impact on cell viability [23]. In a separate report, a concentration effect of lutein on the proliferation and migration of bovine lens epithelial cells was identified [39]. Although differences in tested concentrations and lutein sources make it difficult to draw comparisons between these reports, lutein concentration in our study is correlated with the viability of HLE cells and is a factor which should be considered in future in vitro studies.

When UV exposure was used to induce photo-oxidative stress, all samples experienced a reduction in cell viability (Fig 3). However, this finding was most prominent at higher concentrations of lutein (between 0.25 μg LNP/μL to 0.50 μg LNP/μL) as well as at the highest concentration of BNP (0.50 μg BNP/μL). A reduction in viability at higher lutein concentrations, without UV light exposure, was not unexpected. In one report, lutein did not block loss of cell viability following exposure to 100 μM H_2_O_2_ indicating that some damage triggering subsequent cell death was not blocked by antioxidants; this may be due to a disturbance in other cell signaling pathways ultimately triggering cellular apoptosis [23].

Antioxidants such as lutein scavenge ROS to inhibit their impact on cellular structures [40]. A dose dependent effect on levels of ROS was identified in HLE cells exposed to LNP *in vitro* (Fig 4). At concentrations of 0.25–0.50 μg/μL of LNP, a reduction in ROS was identified compared to BNP. This contrasts with a previous report in which canine lens epithelial cells incubated with lutein demonstrated a significant increase in ROS production [41]. The discrepancy may be related to differences in the employed lutein-delivery platform. For the current report, lutein was prepared and entrapped within negatively charged PLGA nanoparticles. In the previous study, free lutein was solubilized in ethanol and then diluted in DMEM culture medium to a working concentration of 15mg/L [41]. One author found that pre-incubation with lutein at concentrations of 1 μM and 5 μM prior to induction of oxidative stress with peroxide, a dose dependent effect on protein carbonyl and lipid peroxidation occurred [23]. The reduction in ROS identified in our study would explain the finding by Gao et al. and further evaluation of lipid and protein oxidation products in vitro are needed [23]. The lack of reduction in ROS in the BNP group, indicates that the impact on ROS is not related to the PLGA compound or surfactants utilized in the production of NPs.

When cultures were exposed to photo-oxidative stress, an increase in ROS was identified in all groups (Fig 5); however, the increase was not statistically significant. UV has been shown to induce ROS production in a previous report, regardless of treatment, similar to our findings [41]. The lack statistical significance for ROS with UV exposure in this study may be related to the use of a lower energy setting of 0.3 J/cm^2^ as well use of a genetically modified cell line. In the future, other modes of assessing oxidative damage such as evaluation of lipid peroxidation and DNA damage should be explored with this UV model [23].

For the in vivo phase of this study, the ability of topically applied lutein formulations to penetrate the eye and attenuate selenite-induced cataract formation in Wistar rats were evaluated. Sodium-selenite injection has been used to induce cataracts in young rats (<16 days) with morphological similarities to human cataracts, and thus it was selected as the model utilized [18, 42].

Since oxidative stress plays a role in the development of both the selenite in vivo model and naturally acquired cataract formation, the ability of topically applied LNP compared to free lutein formulas, to reduce cataract formation was evaluated in vivo. Given the nature of the eye, it is difficult to replicate the static and dynamic barriers of the eye in an in vitro model. Currently, more than 90% of ophthalmic medications are formulated as an eye drop or topical ointment [43]. Unfortunately, significant barriers to ocular drug delivery are encountered following topical application of these products. These barriers include precorneal factors such as the elimination of applied products though blinking, the dilution of medications by the precorneal tear film, short contact time in the conjunctival fornix, as well as the inherent physical barriers of the cornea. Physical barriers include a unique arrangement of hydrophilic-hydrophobic corneal layers as well as tight junctions of the corneal epithelium. The pore size of the corneal epithelium is approximately 2 nm with low pore density compared to the conjunctiva [44, 45]. These barriers result in a significant impediment to topically applied drugs based on lipophilicity, solubility, size shape and charge of the compound.

To reduce rapid elimination of topically applied nanoparticles, tested nanoparticles were embedded in a thermosensitive hydrogel which solidifies upon contact with the warm corneal surface, increasing the corneal residence time, and facilitating trans-corneal permeation [12]. Examination of all animals by slit lamp biomicroscopy showed no evidence of keratitis or anterior uveitis associated with topical treatment. Due to the presence of tight junctions in the corneal epithelium, the tested nanoparticles primarily cross the epithelial layer via transcellular permeation. Other potential routes of entry of topically applied products into the intraocular compartment include the transconjunctival and transscleral routes; however, these routes are primarily utilized by compounds of a hydrophilic nature [46, 47]. While the nanoparticles are composed of lipophilic polymers, they are nevertheless suspended in an aqueous medium with the aid of surfactant coatings. Whether the entire nanoparticle can permeate through the cornea into the anterior chamber is unclear, however lutein was identified in aqueous aspirates of treated eyes and absent in control samples (S1 and S2 Figs). Additional evaluation by targeted LC-MS/MS identified spectra consistent with lutein in all lens samples tested (S3 and S4 Figs). In contrast with the aqueous humor results, finding low level of lutein within all lenses is likely due to lutein obtained through dietary sources. In other species, lutein is normally found within the lens and further investigation in rodent models is warranted [48, 49]. Despite this hypothesis, compared to controls, higher calculated AUC was identified in lenses of treated eyes and most prominent at the one-hour time point. Given the detectable lutein in the aqueous humor the authors theorize that lutein present in topical LNP penetrate the anterior chamber and is capable of diffusing to the lens in rodents. Due to the small volume of samples available for analysis, lutein was detectable, however, values were below the limit of quantification.

The in vivo model identified a significant reduction in cataract severity for animals receiving 1278 μg/mL lutein (Fig 6). This finding supports the hypothesis that lutein in the tested nanoparticle formulation can reach the intraocular compartment and counteract the cataract-inducing mechanisms of sodium selenite. The cause for the observed reduction in cataract severity at 1,278 μg/mL compared to a higher lutein nanoparticle concentration of 2130 μg/mL is unclear; a similar trend was reported in a previous study for PLGA nanoparticles at the same tested concentrations [12]. Lutein’s mechanism of protection against selenite-induced cataracts likely involves the loss of calcium homeostasis in the lenses of rats after administering selenite, possibly by protecting ion channels and Ca^2+^_ ATPase from oxidative damage [18, 50]. Typically, selenite causes rapid accumulation of calcium in lens epithelial cells, resulting in the activation of the m-Calpain protease and subsequent cleavage of crystallin proteins, causing the loss of lens transparency [18]; further evaluation of this theory in vitro would be needed to confirm this mechanism.

Our results differed from a previous study in that cataract scores were much lower, and a significant impact of high dose lutein treatment was also identified in that report [12]. There are several possible causes for these differences. The strain of rat used in this report differed from the previous study as well as differences in study design including the use of a different cataract grading system and use of pharmacological dilation performed prior to cataract grading.

Of additional consideration for both reports, some variability between batches of lutein may have occurred although the same source of lutein was utilized. Lutein and its isomers contain a polyene chain with numerous conjugated double bonds and a hydroxyl group present at each terminal ring. Lutein and zeaxanthin are positional isomers which may be a factor in the biological functioning of the carotenoid depending on the orientation of the molecule as it spans cellular membranes [34, 51]. Carotenoids are also susceptible to oxidative changes as well as changes in their crystalloid matrix. These lipophilic pigments have been shown to form supramolecular aggregates of which this conformation may affect their physical properties and functions [51, 52]. Emulsification has been reported to affect their crystalline structure [52]. Therefore, emulsification of carotenoids and the conditions of processing and drying could result in difference in function based on batch sources and changes in crystalline structure of lutein may be a factor in the differences observed [52]. Analysis of crystalline carotenoids via X-ray diffraction or UV/Vis spectroscopy would be needed to confirm this hypothesis [52].

Since oxidative stress plays a role in the development of selenite and age-related cataracts, results of the present study suggest the potential of topically applied, lutein-loaded nanoparticles to reduce the progression of age-related cataracts [12]. However, there are several limitations to this study which require consideration.

Oxidative stress plays a role in the development of selenite and age-related cataracts, therefore this study focused on cataracts developing secondary to oxidative stress to evaluate treatment with an antioxidant compound. Based on this focus, findings are limited to cataracts secondary to oxidative stress. Additionally, only reactive oxygen species development was tested in this study. To complete the evaluation of topical antioxidant therapy on ocular oxidative stress, future studies investigating endogenous lens antioxidant systems are needed.

While selenite-induced cataract formation in young rats provides a convenient model to test therapies for cataract prevention, the rat eye is substantially smaller than eyes of humans and larger animal models. Eye size is considered a significant variable when evaluating the distribution and pharmacokinetics of topically applied drugs [47]. The results in this report indicate the successful delivery of topically applied lutein to the anterior segment of rats and augmentation in cataract formation. However, further testing is necessary to determine if a similar effect is seen in the eyes larger species and ultimately leads to detectable lens lutein concentrations. Long-term studies evaluating nanoparticle stability, efficacy, and tolerance after topical application would be required prior to use in clinical patients.

## Conclusion

In this study, LNP had a minimal impact on HLE cell viability during a short exposure timeframe (24 h) at concentrations less than 0.20 μg/μl. A significant reduction in ROS development occurred at concentrations from 0.25 μg LNP/μL to 0.50 μg LNP/μL, compared to BNP. In vivo, the ability of topically administered lutein to reach the anterior segment of rats and protect against cataract-forming mechanisms of selenite was demonstrated. Cataract development in rat pups was attenuated by the once daily application of lutein loaded PLGA nanoparticles embedded in a thermosensitive hydrogel. The most effective formulation tested was demonstrated to be 1278 μg of lutein/mL. This study supports the hypothesis that the topical application of lutein loaded, polymeric nanoparticles may be an effective therapy for cataract prevention.

## Supporting information

S1 Fig Chromatograms of aqueous humor samples. Extracted ion chromatogram of lutein from tested aqueous humor samples. Lutein was detected in both the one-hour and two-hour treated samples (Treatment 1 = treatment, 1-hour time point; Treatment 2 = treatment, 2-hour time point) and not detected in control samples (Control 1 = control, 1-hour time point; Control 2 = control, 2-hour time point). Difference in retention times were < 0.1 min.
(TIFF)

S2 Fig Spectra of aqueous humor samples. Averaged full spectra from tested aqueous humor samples compared to lutein standard. Although controls have traces of the peak, they were well below the limit of detection and outside the 5 part per million error used as tolerance. Treatment 1-hour and Treatment 2-hour had ~10x the signal of the controls (below LOQ).
(TIFF)

S3 Fig Chromatograms of lens samples collected one-hour following treatment. Extracted chromatogram for lens analyte evaluated for lutein by LC-MS/MS. Extracted ion chromatogram utilize the 338 and 476 fragments associated with lutein for lens sample analysis. Sample T1- Treated lens sample, 1 hour. Percent intensity is indicated on the Y-axis and Time (min) is indicated on the X-axis. Horizontal reference line included with each chromatogram to demonstrate intensity of lowest lutein calibration point for relative intensity comparison.
(TIFF)

S4 Fig Spectra of lens samples collected one-hour following treatment. Mass spectra of lens analyte evaluated for lutein by LC-MS/MS. Sample T1- Treated lens sample, 1 hour. Percent intensity is indicated on the Y-axis and ion (m/z) is on X-axis. Characteristic fragments are highlighted and compared to 0.2ng lutein calibration.
(TIFF)

S5 Fig Chromatograms of lens samples collected two-hours following treatment. Extracted chromatogram for lens analyte evaluated for lutein by LC-MS/MS. Extracted ion chromatogram utilize the 338 and 476 fragments associated with lutein for lens sample analysis. Sample T2- Treated lens samples, 2 hours. Percent intensity is indicated on the Y-axis and Time (min) is indicated on the X-axis. Horizontal reference line included with each chromatogram to demonstrate intensity of lowest lutein calibration point for relative intensity comparison.
(PDF)

S6 Fig Spectra of lens samples collected two-hours following treatment. Example mass spectra of lens analyte evaluated for lutein by LC-MS/MS. Sample T2- Treated lens sample, 2 hours. Percent intensity is indicated on the Y-axis and ion (m/z) is on X-axis. Characteristic fragments are highlighted and compared to 0.2ng lutein calibration. Characteristic fragments are indicated.
(PDF)

S7 Fig Spectra of lens control samples collected two-hours following application of control. Example mass spectra of lens analyte evaluated for lutein by LC-MS/MS. Sample C2- Control lens sample, 2 hours. Percent intensity is indicated on the Y-axis and ion (m/z) is on X-axis. Characteristic fragments are highlighted and compared to 0.2ng lutein calibration. Characteristic fragments are indicated.
(PDF)
