# Supplementary figures and images for: The impact of lutein-loaded poly(lactic-co-glycolic acid) nanoparticles following topical application: An in vitro and in vivo study
Source: PLoS One. 2024 Aug 1;19(8):e0306640. doi: 10.1371/journal.pone.0306640 (PMC11293729; doi:10.1371/journal.pone.0306640)

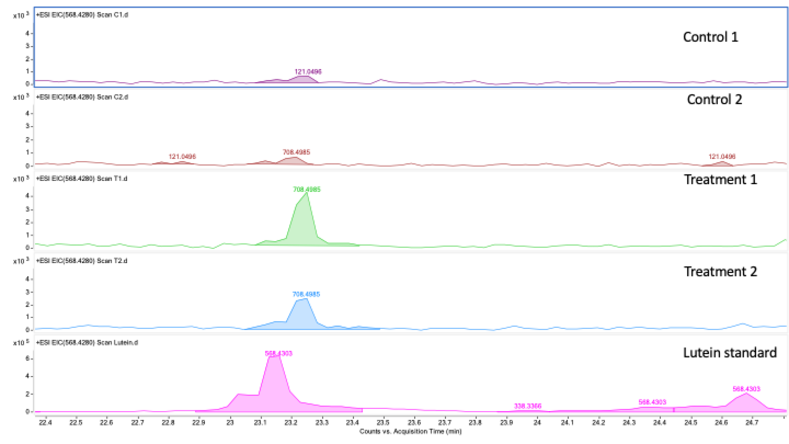

Supplement: S1 Fig — Extracted ion chromatogram of lutein from tested aqueous humor samples. Lutein was detected in both the one-hour and two-hour treated samples (Treatment 1 = treatment, 1-hour time point; Treatment 2 = treatment, 2-hour time point) and not detected in control samples (Control 1 = control, 1-hour time point; Control 2 = control, 2-hour time point). Difference in retention times were < 0.1 min. (TIFF) [file pone.0306640.s001.tiff]

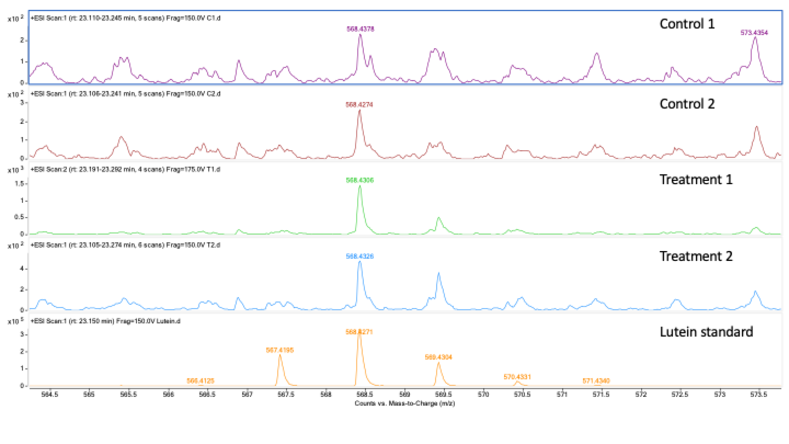

Supplement: S2 Fig — Averaged full spectra from tested aqueous humor samples compared to lutein standard. Although controls have traces of the peak, they were well below the limit of detection and outside the 5 part per million error used as tolerance. Treatment 1-hour and Treatment 2-hour had ~10x the signal of the controls (below LOQ). (TIFF) [file pone.0306640.s002.tiff]

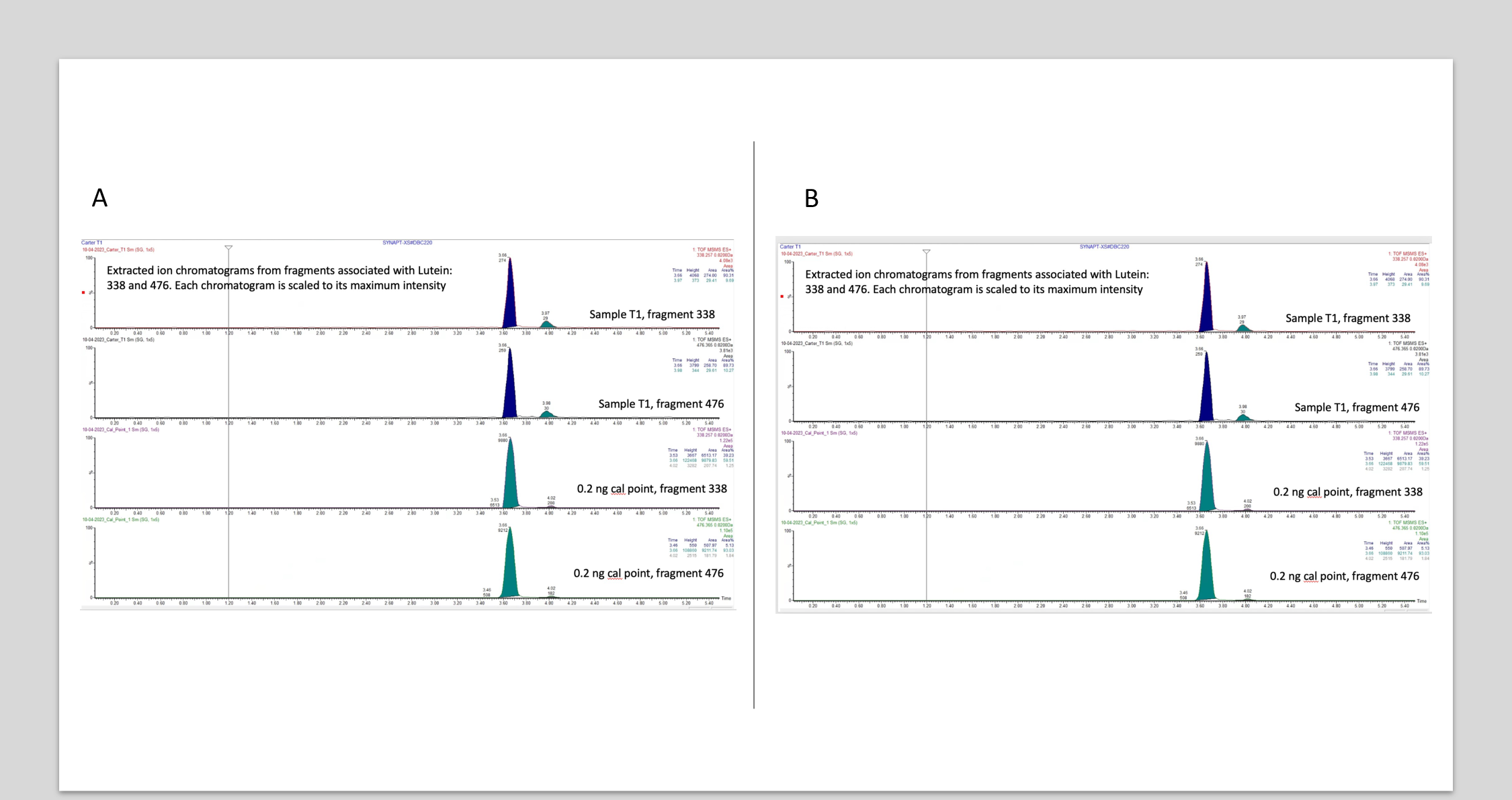

Supplement: S3 Fig — Extracted chromatogram for lens analyte evaluated for lutein by LC-MS/MS. Extracted ion chromatogram utilize the 338 and 476 fragments associated with lutein for lens sample analysis. Sample T1- Treated lens sample, 1 hour. Percent intensity is indicated on the Y-axis and Time (min) is indicated on the X-axis. Horizontal reference line included with each chromatogram to demonstrate intensity of lowest lutein calibration point for relative intensity comparison. (TIFF) [file pone.0306640.s003.tiff]

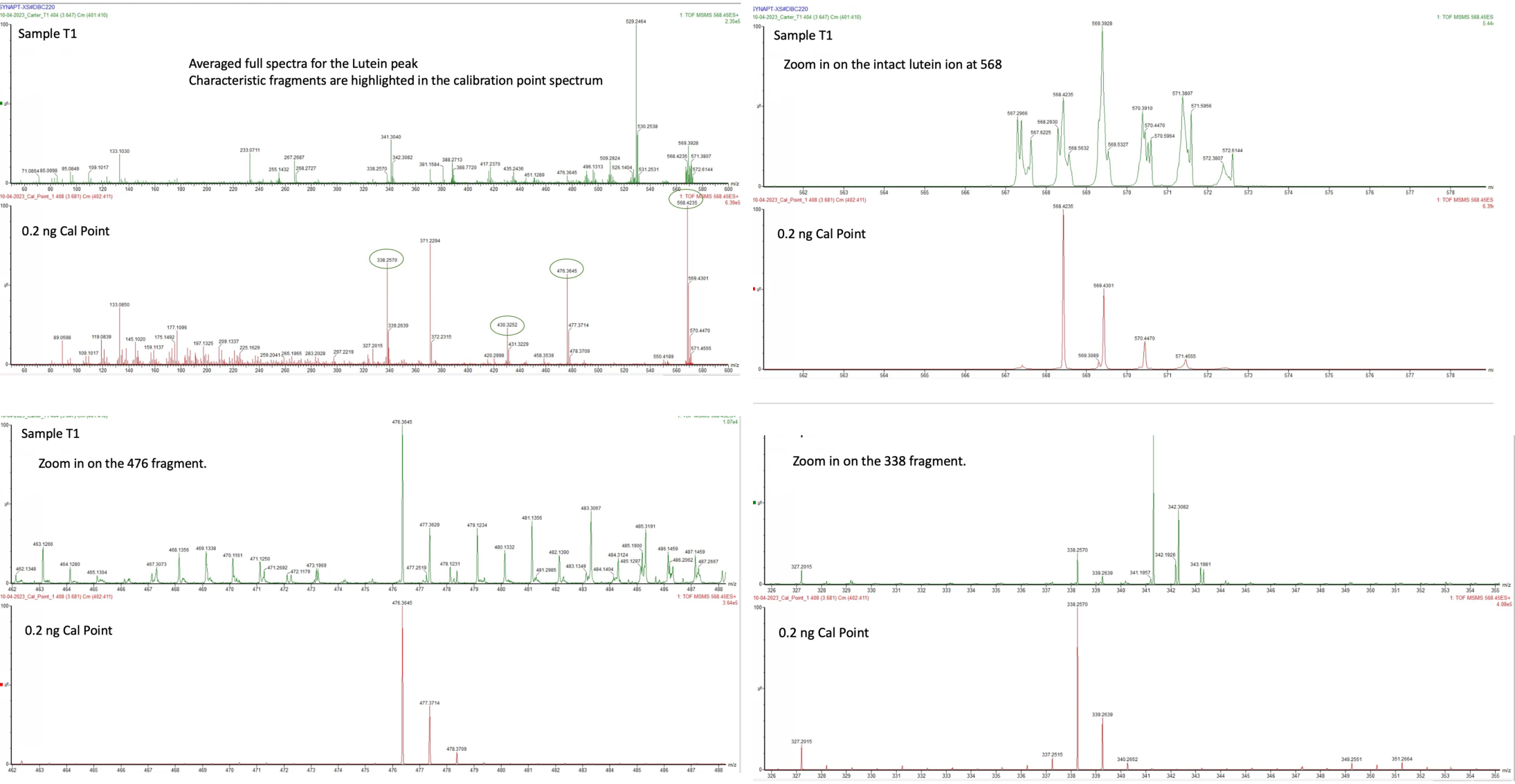

Supplement: S4 Fig — Mass spectra of lens analyte evaluated for lutein by LC-MS/MS. Sample T1- Treated lens sample, 1 hour. Percent intensity is indicated on the Y-axis and ion (m/z) is on X-axis. Characteristic fragments are highlighted and compared to 0.2ng lutein calibration. (TIFF) [file pone.0306640.s004.tiff]
