# Supplementary material for: The impact of lutein-loaded poly(lactic-co-glycolic acid) nanoparticles following topical application: An in vitro and in vivo study
Source: PLoS One. 2024 Aug 1;19(8):e0306640. doi: 10.1371/journal.pone.0306640 (PMC11293729; doi:10.1371/journal.pone.0306640)
Supplement: S5 Fig — Extracted chromatogram for lens analyte evaluated for lutein by LC-MS/MS. Extracted ion chromatogram utilize the 338 and 476 fragments associated with lutein for lens sample analysis. Sample T2- Treated lens samples, 2 hours. Percent intensity is indicated on the Y-axis and Time (min) is indicated on the X-axis. Horizontal reference line included with each chromatogram to demonstrate intensity of lowest lutein calibration point for relative intensity comparison. (PDF) [file pone.0306640.s005.pdf]

Sample T2

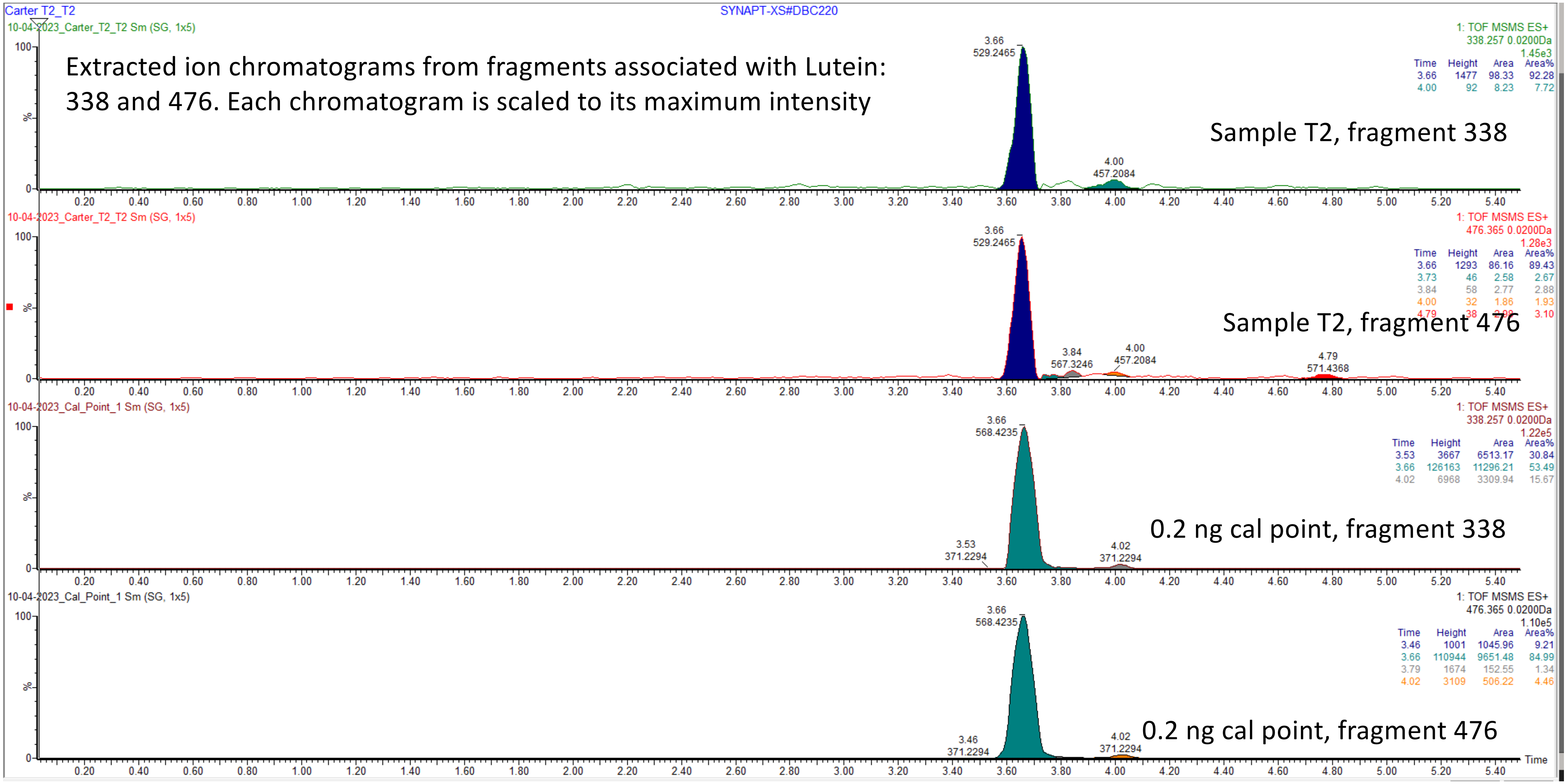
Carter T2_T2
SYNAPT-XS#DBC220
10-04-2023_Carter_T2_T2 Sm (SG, 1x5)
1: TOF MSMS ES+
338.257 0.0200Da
1.45e3
Extracted ion chromatograms from fragments associated with Lutein: 338 and 476. Each chromatogram is scaled to its maximum intensity
Time Height Area Area%
3.66 1477 98.33 92.28
4.00 92 8.23 7.72
Sample T2, fragment 338
10-04-2023_Carter_T2_T2 Sm (SG, 1x5)
1: TOF MSMS ES+
476.365 0.0200Da
1.28e3
Time Height Area Area%
3.66 1293 86.16 89.43
3.73 46 2.58 2.67
3.84 58 2.77 2.88
4.00 32 1.86 1.93
Sample T2, fragment 476
10-04-2023_Cal_Point_1 Sm (SG, 1x5)
1: TOF MSMS ES+
338.257 0.0200Da
1.22e5
Time Height Area Area%
3.53 3667 6513.17 30.84
3.66 126163 11296.21 53.49
4.02 6968 3309.94 15.67
0.2 ng cal point, fragment 338
10-04-2023_Cal_Point_1 Sm (SG, 1x5)
1: TOF MSMS ES+
476.365 0.0200Da
1.10e5
Time Height Area Area%
3.46 1001 1045.96 9.21
3.66 110944 9651.48 84.99
3.79 1674 152.55 1.34
4.02 3109 506.22 4.46
0.2 ng cal point, fragment 476
Time

Sample T2

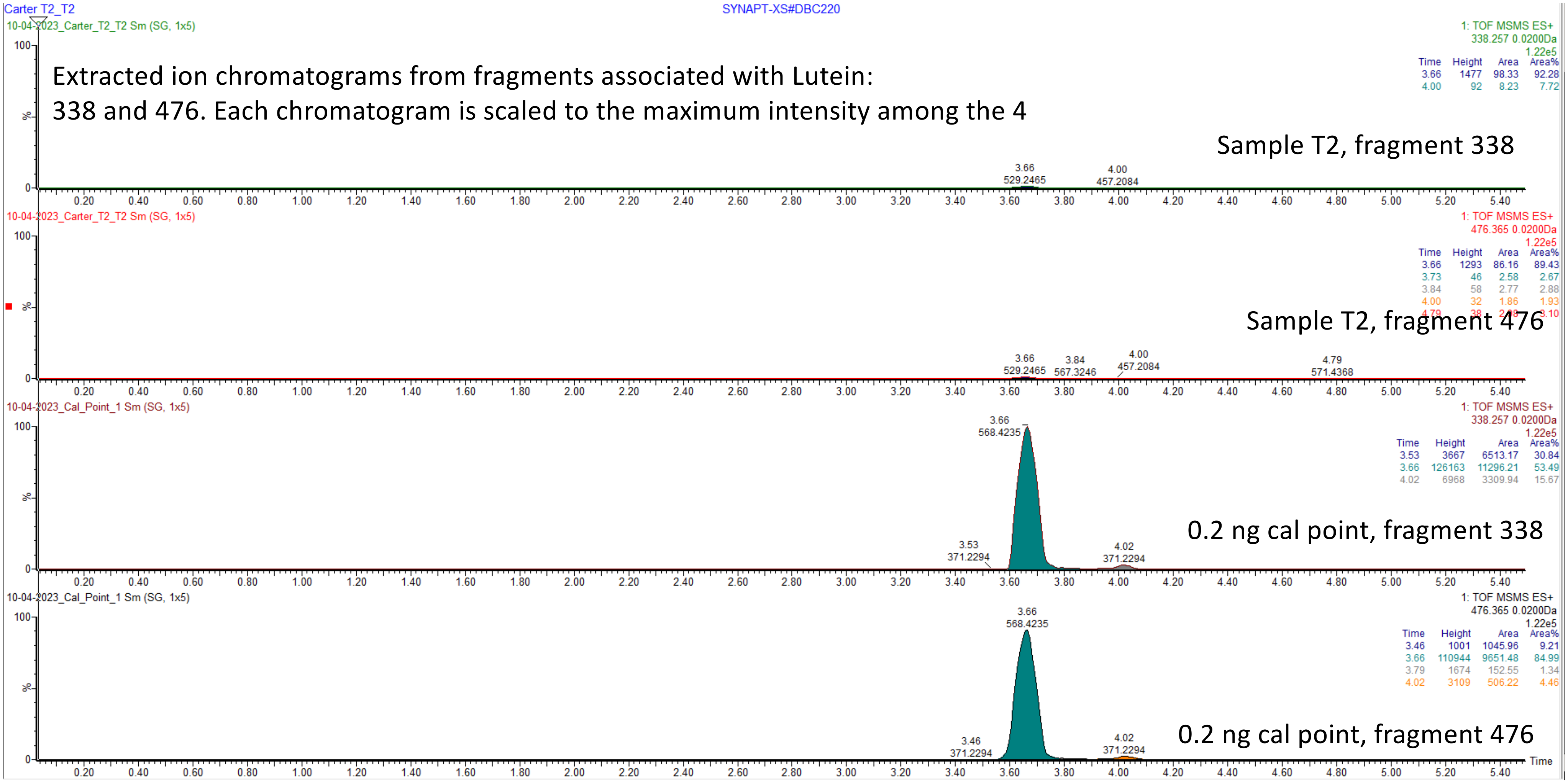

Carter T2_T2
SYNAPT-XS#DBC220
Extracted ion chromatograms from fragments associated with Lutein:
338 and 476. Each chromatogram is scaled to the maximum intensity among the 4
10-04-2023_Carter_T2_T2 Sm (SG, 1x5)
1: TOF MSMS ES+
338.257 0.0200Da
1.22e5
Time Height Area Area%
3.66 1477 98.33 92.28
4.00 92 8.23 7.72
Sample T2, fragment 338
3.66 529.2465
4.00 457.2084
10-04-2023_Carter_T2_T2 Sm (SG, 1x5)
1: TOF MSMS ES+
476.365 0.0200Da
1.22e5
Time Height Area Area%
3.66 1293 86.16 89.43
3.73 46 2.58 2.67
3.84 58 2.77 2.88
4.00 32 1.86 1.93
Sample T2, fragment 476
3.66 529.2465
3.84 567.3246
4.00 457.2084
4.79 571.4368
10-04-2023_Cal_Point_1 Sm (SG, 1x5)
1: TOF MSMS ES+
338.257 0.0200Da
1.22e5
Time Height Area Area%
3.53 3667 6513.17 30.84
3.66 126163 11296.21 53.49
4.02 6968 3309.94 15.67
3.66 568.4235
3.53 371.2294
4.02 371.2294
0.2 ng cal point, fragment 338
10-04-2023_Cal_Point_1 Sm (SG, 1x5)
1: TOF MSMS ES+
476.365 0.0200Da
1.22e5
Time Height Area Area%
3.46 1001 1045.96 9.21
3.66 110944 9651.48 84.99
3.79 1674 152.55 1.34
4.02 3109 506.22 4.46
3.66 568.4235
3.46 371.2294
4.02 371.2294
0.2 ng cal point, fragment 476
Time
