# Supplementary material for: The impact of lutein-loaded poly(lactic-co-glycolic acid) nanoparticles following topical application: An in vitro and in vivo study
Source: PLoS One. 2024 Aug 1;19(8):e0306640. doi: 10.1371/journal.pone.0306640 (PMC11293729; doi:10.1371/journal.pone.0306640)
Supplement: S6 Fig — Example mass spectra of lens analyte evaluated for lutein by LC-MS/MS. Sample T2- Treated lens sample, 2 hours. Percent intensity is indicated on the Y-axis and ion (m/z) is on X-axis. Characteristic fragments are highlighted and compared to 0.2ng lutein calibration. Characteristic fragments are indicated. (PDF) [file pone.0306640.s006.pdf]

Sample T2

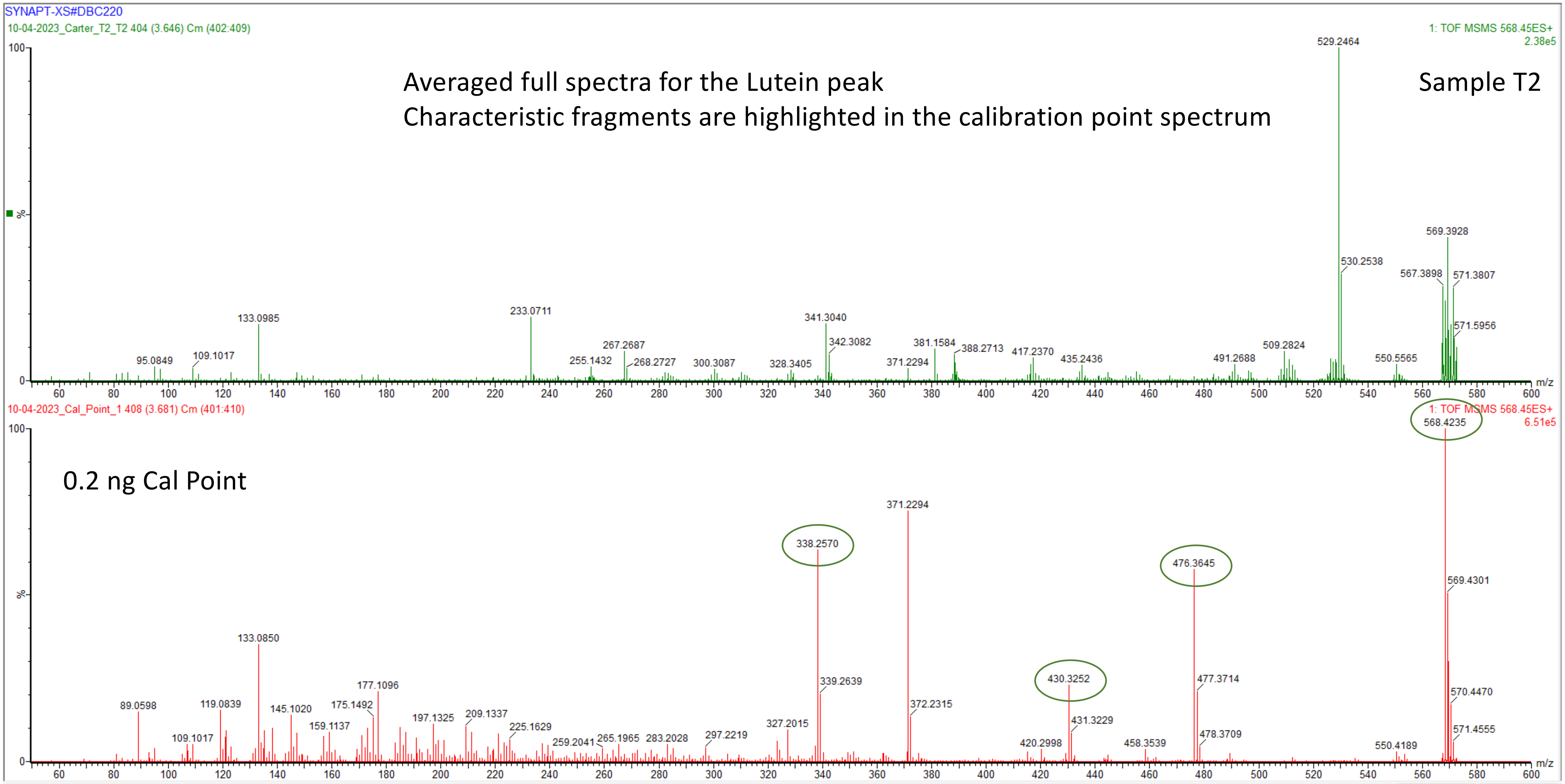
SYNAPT-XS#DBC220
10-04-2023_Carter_T2_T2 404 (3.646) Cm (402:409)
1: TOF MSMS 568.45ES+
2.38e5
Averaged full spectra for the Lutein peak
Characteristic fragments are highlighted in the calibration point spectrum
Sample T2
529.2464
569.3928
530.2538
567.3898
571.3807
571.5956
133.0985
233.0711
341.3040
342.3082
381.1584
388.2713
417.2370
435.2436
267.2687
255.1432
268.2727
300.3087
328.3405
371.2294
95.0849
109.1017
491.2688
509.2824
550.5565
10-04-2023_Cal_Point_1 408 (3.681) Cm (401:410)
1: TOF MSMS 568.45ES+
6.51e5
568.4235
0.2 ng Cal Point
371.2294
338.2570
476.3645
569.4301
133.0850
339.2639
430.3252
477.3714
570.4470
177.1096
89.0598
119.0839
145.1020
175.1492
372.2315
159.1137
197.1325
209.1337
225.1629
327.2015
431.3229
571.4555
109.1017
259.2041
265.1965
283.2028
297.2219
420.2998
458.3539
478.3709
550.4189
m/z
%

Sample T2

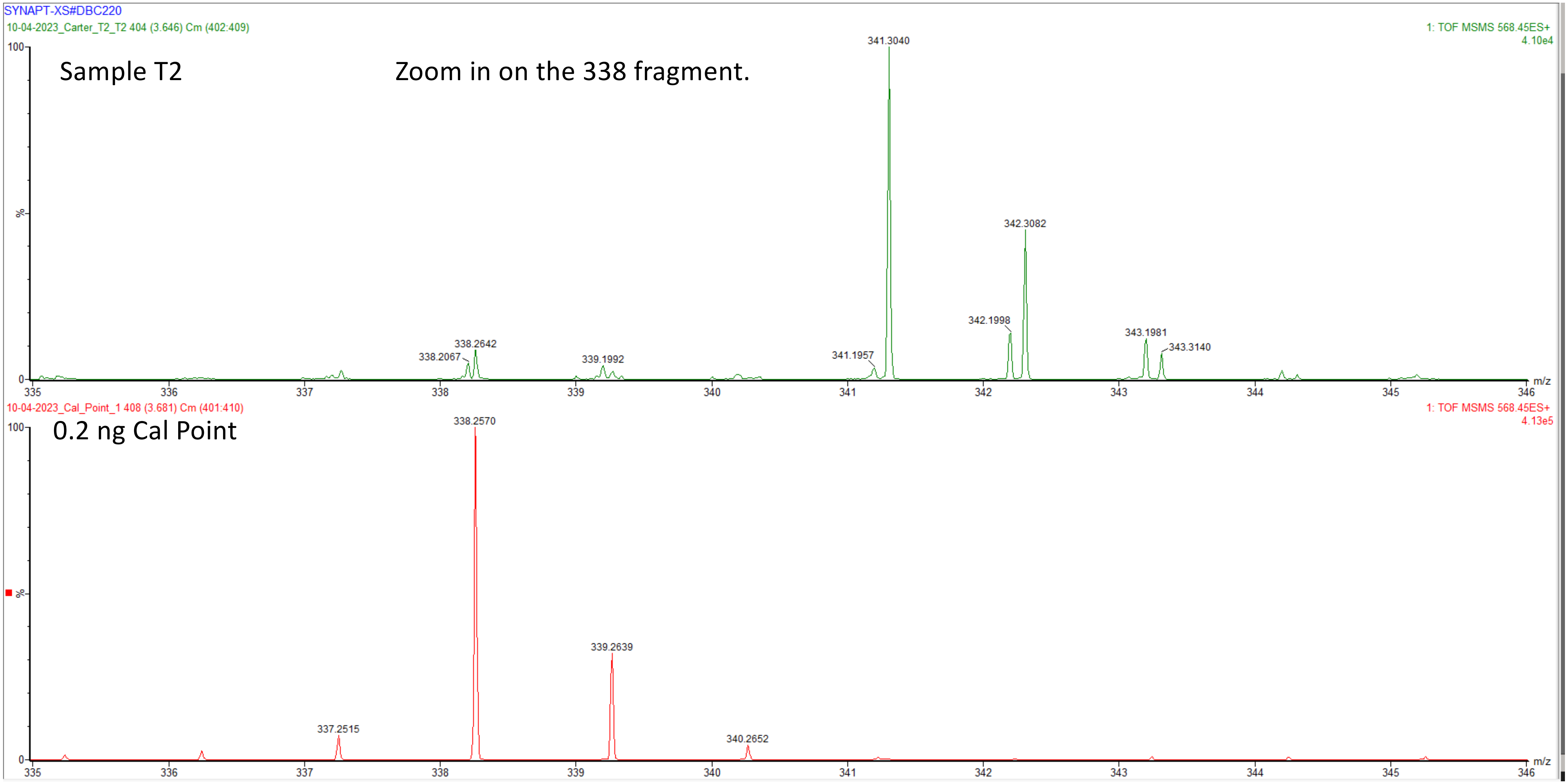

SYNAPT-XS#DBC220
10-04-2023_Carter_T2_T2 404 (3.646) Cm (402:409)
1: TOF MSMS 568.45ES+
4.10e4
Sample T2
Zoom in on the 338 fragment.
341.3040
342.3082
342.1998
343.1981
343.3140
338.2642
338.2067
339.1992
341.1957
10-04-2023_Cal_Point_1 408 (3.681) Cm (401:410)
1: TOF MSMS 568.45ES+
4.13e5
0.2 ng Cal Point
338.2570
339.2639
337.2515
340.2652
m/z
%

Sample T2

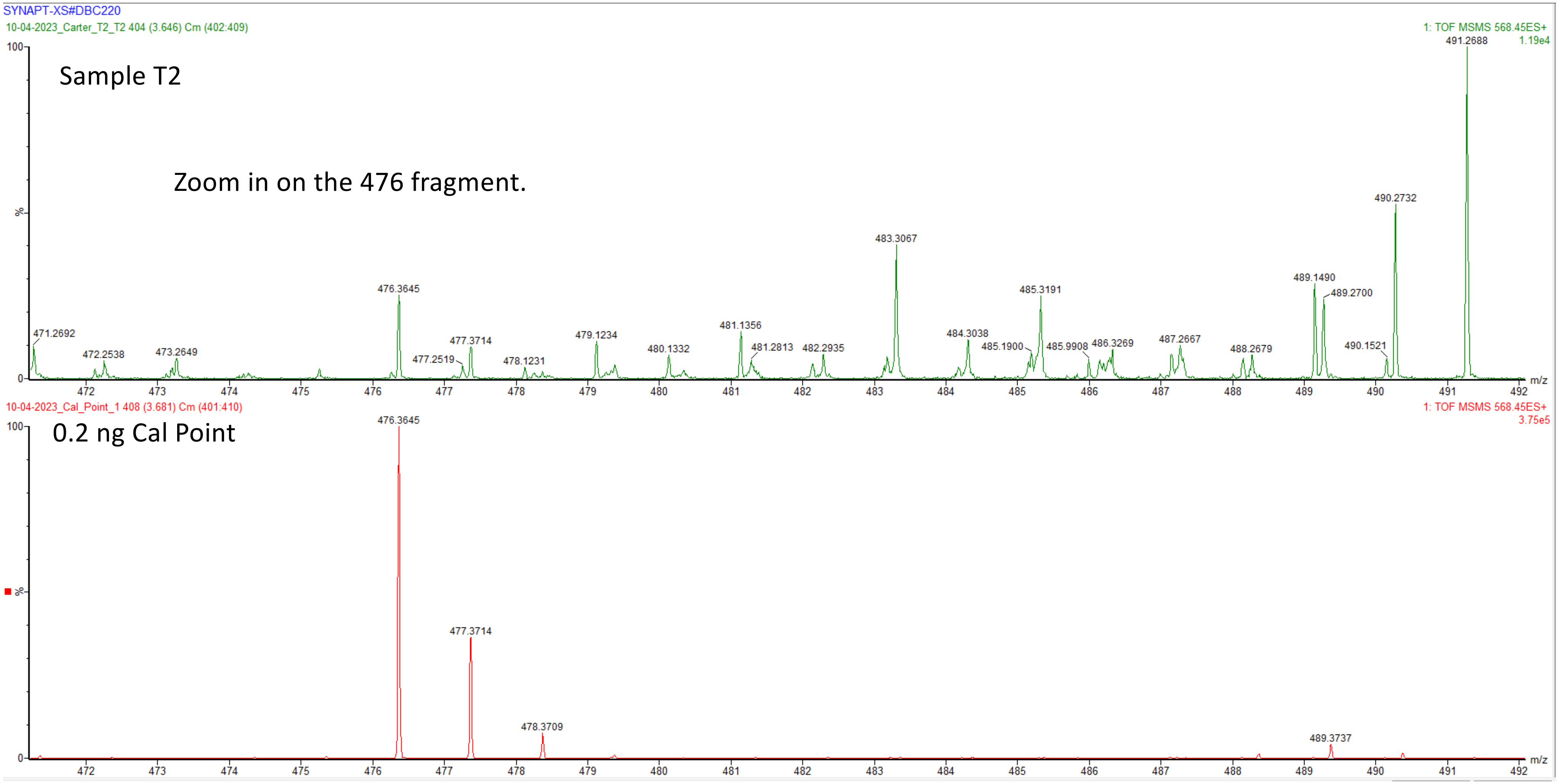

SYNAPT-XS#DBC220
10-04-2023_Carter_T2_T2 404 (3.646) Cm (402:409)
1: TOF MSMS 568.45ES+
1.19e4
Sample T2
Zoom in on the 476 fragment.
491.2688
490.2732
483.3067
489.1490
489.2700
476.3645
485.3191
481.1356
471.2692
477.3714
479.1234
484.3038
487.2667
472.2538
473.2649
477.2519
478.1231
480.1332
481.2813
482.2935
485.1900
485.9908
486.3269
488.2679
490.1521
10-04-2023_Cal_Point_1 408 (3.681) Cm (401:410)
1: TOF MSMS 568.45ES+
3.75e5
0.2 ng Cal Point
476.3645
477.3714
478.3709
489.3737
100
%
0
m/z
472
473
474
475
476
477
478
479
480
481
482
483
484
485
486
487
488
489
490
491
492

Sample T2

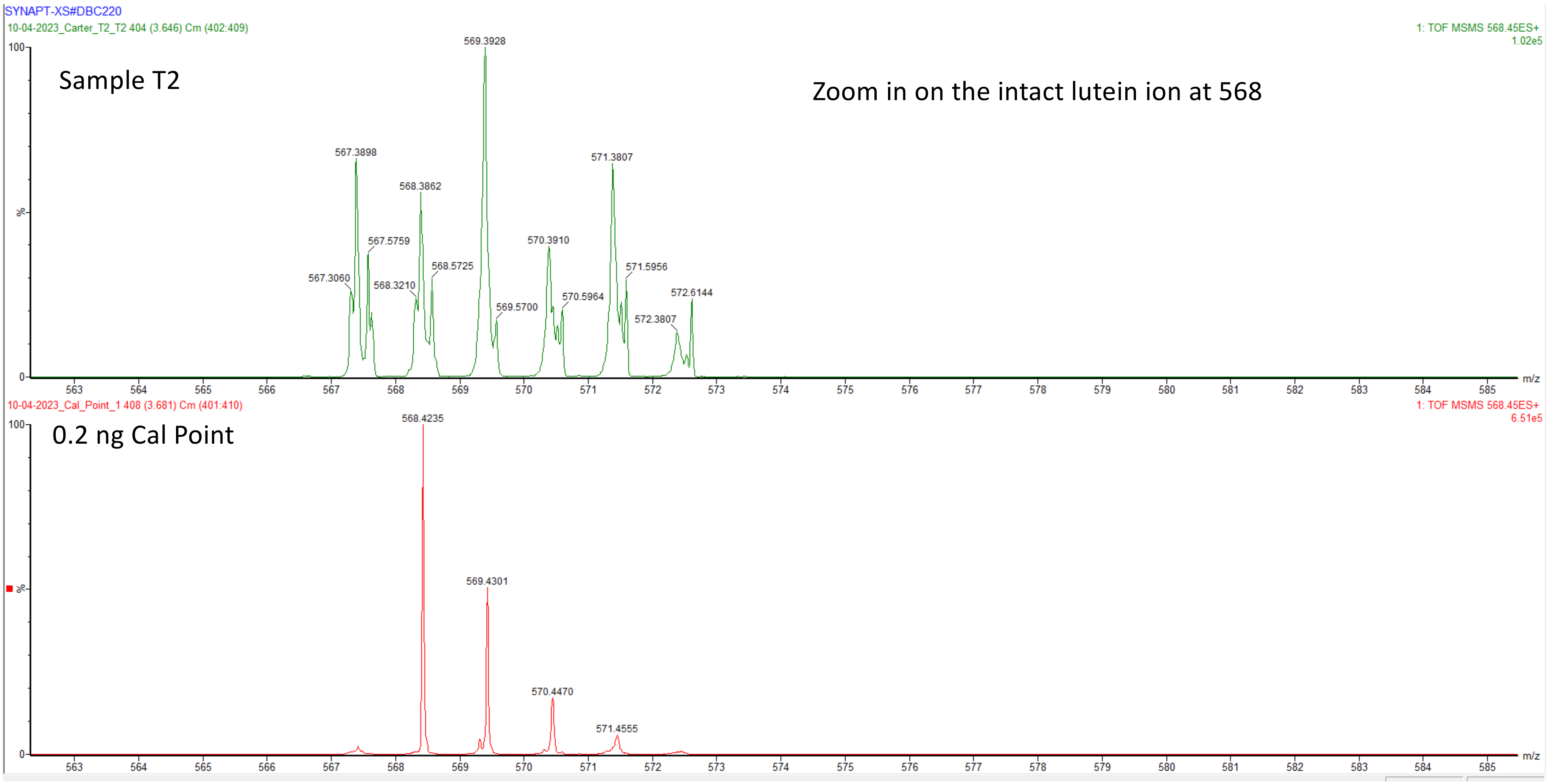

SYNAPT-XS#DBC220
10-04-2023_Carter_T2_T2 404 (3.646) Cm (402:409)
1: TOF MSMS 568.45ES+
1.02e5
Sample T2
Zoom in on the intact lutein ion at 568
569.3928
567.3898
571.3807
568.3862
567.5759
570.3910
567.3060
568.3210
568.5725
571.5956
569.5700
570.5964
572.6144
572.3807
100
%
0
563 564 565 566 567 568 569 570 571 572 573 574 575 576 577 578 579 580 581 582 583 584 585
m/z
10-04-2023_Cal_Point_1 408 (3.681) Cm (401:410)
1: TOF MSMS 568.45ES+
6.51e5
0.2 ng Cal Point
568.4235
569.4301
570.4470
571.4555
