# Supplementary material for: The impact of lutein-loaded poly(lactic-co-glycolic acid) nanoparticles following topical application: An in vitro and in vivo study
Source: PLoS One. 2024 Aug 1;19(8):e0306640. doi: 10.1371/journal.pone.0306640 (PMC11293729; doi:10.1371/journal.pone.0306640)
Supplement: S7 Fig — Example mass spectra of lens analyte evaluated for lutein by LC-MS/MS. Sample C2- Control lens sample, 2 hours. Percent intensity is indicated on the Y-axis and ion (m/z) is on X-axis. Characteristic fragments are highlighted and compared to 0.2ng lutein calibration. Characteristic fragments are indicated. (PDF) [file pone.0306640.s007.pdf]

Control 2

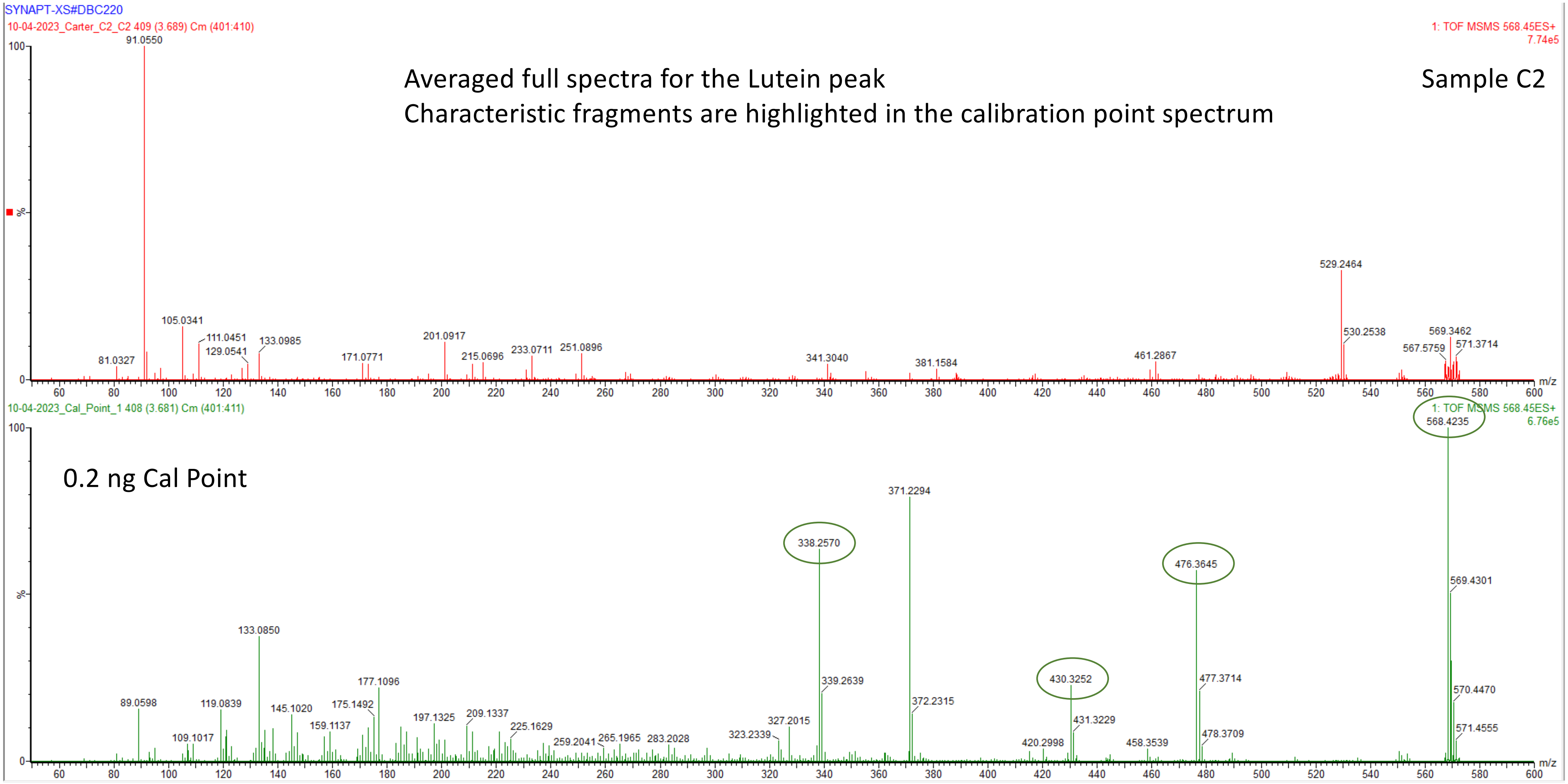
SYNAPT-XS#DBC220
10-04-2023_Carter_C2_C2 409 (3.689) Cm (401:410)
1: TOF MSMS 568.45ES+
7.74e5
Averaged full spectra for the Lutein peak
Characteristic fragments are highlighted in the calibration point spectrum
Sample C2
91.0550
105.0341
111.0451
129.0541
133.0985
81.0327
171.0771
201.0917
215.0696
233.0711
251.0896
341.3040
381.1584
461.2867
529.2464
530.2538
567.5759
569.3462
571.3714
m/z
10-04-2023_Cal_Point_1 408 (3.681) Cm (401:411)
1: TOF MSMS 568.45ES+
6.76e5
0.2 ng Cal Point
568.4235
371.2294
338.2570
476.3645
569.4301
133.0850
177.1096
89.0598
119.0839
145.1020
175.1492
159.1137
109.1017
197.1325
209.1337
225.1629
259.2041
265.1965
283.2028
323.2339
327.2015
339.2639
372.2315
430.3252
431.3229
420.2998
458.3539
477.3714
478.3709
570.4470
571.4555
m/z

Control 2

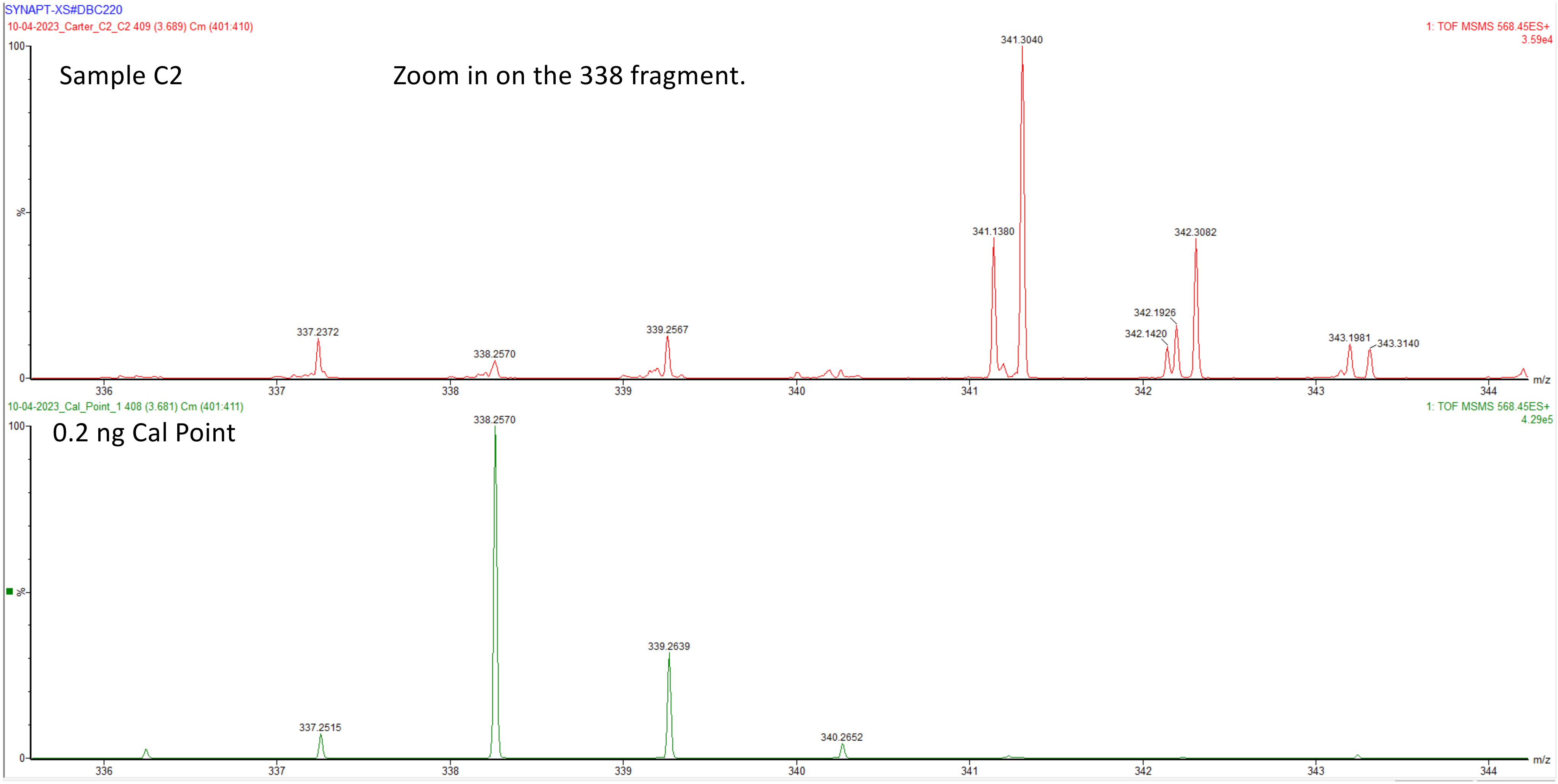

SYNAPT-XS#DBC220
10-04-2023_Carter_C2_C2 409 (3.689) Cm (401:410)
1: TOF MSMS 568.45ES+
3.59e4
Sample C2
Zoom in on the 338 fragment.
341.3040
341.1380
342.3082
342.1926
342.1420
343.1981
343.3140
337.2372
338.2570
339.2567
10-04-2023_Cal_Point_1 408 (3.681) Cm (401:411)
1: TOF MSMS 568.45ES+
4.29e5
0.2 ng Cal Point
338.2570
339.2639
337.2515
340.2652
m/z

Control 2

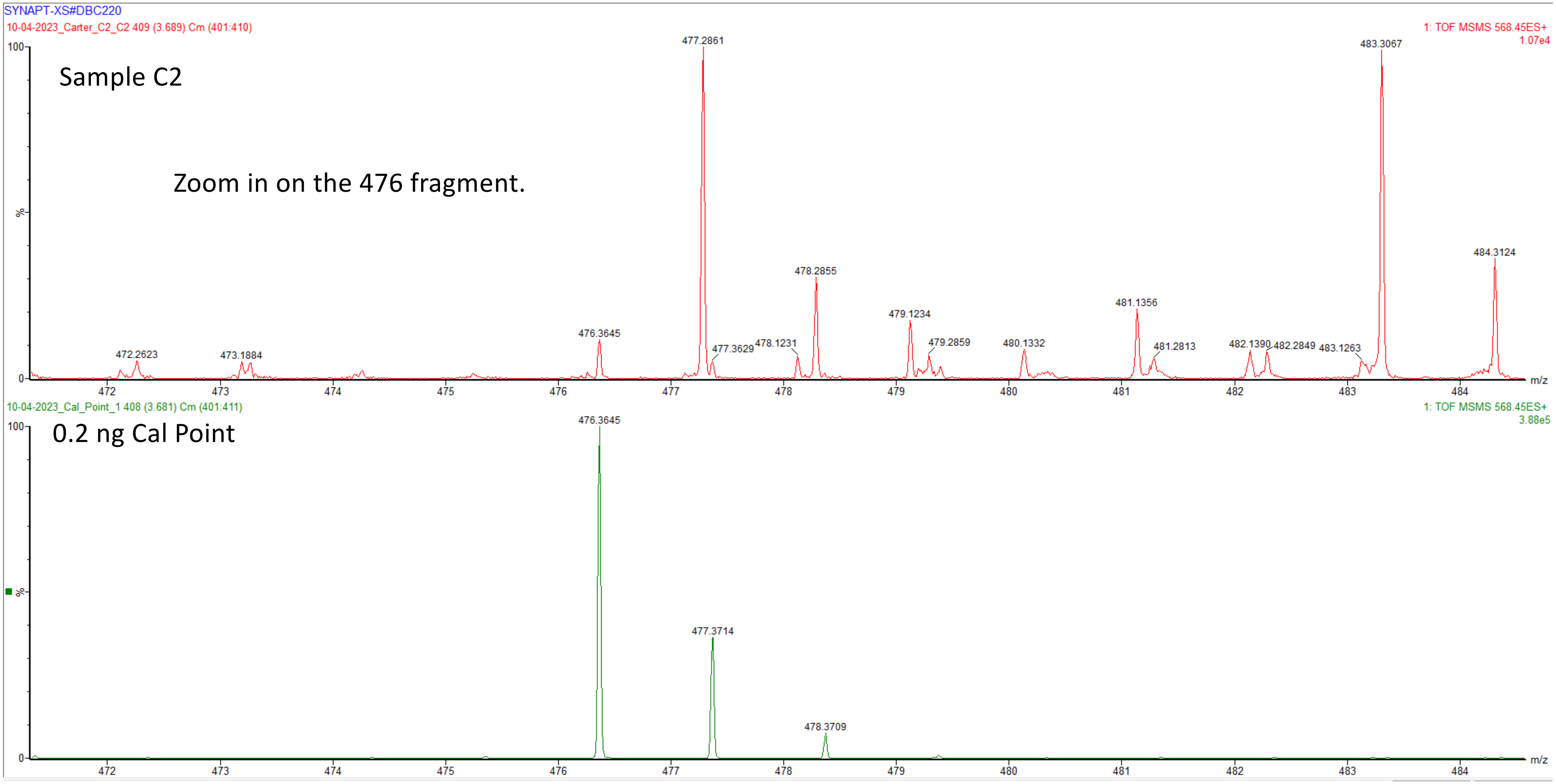
SYNAPT-XS#DBC220
10-04-2023_Carter_C2_C2 409 (3.689) Cm (401:410)
1: TOF MSMS 568.45ES+
1.07e4
Sample C2
Zoom in on the 476 fragment.
477.2861
483.3067
484.3124
478.2855
481.1356
479.1234
476.3645
477.3629
478.1231
479.2859
480.1332
481.2813
482.1390
482.2849
483.1263
472.2623
473.1884
10-04-2023_Cal_Point_1 408 (3.681) Cm (401:411)
1: TOF MSMS 568.45ES+
3.88e5
0.2 ng Cal Point
476.3645
477.3714
478.3709
m/z
%

Control 2

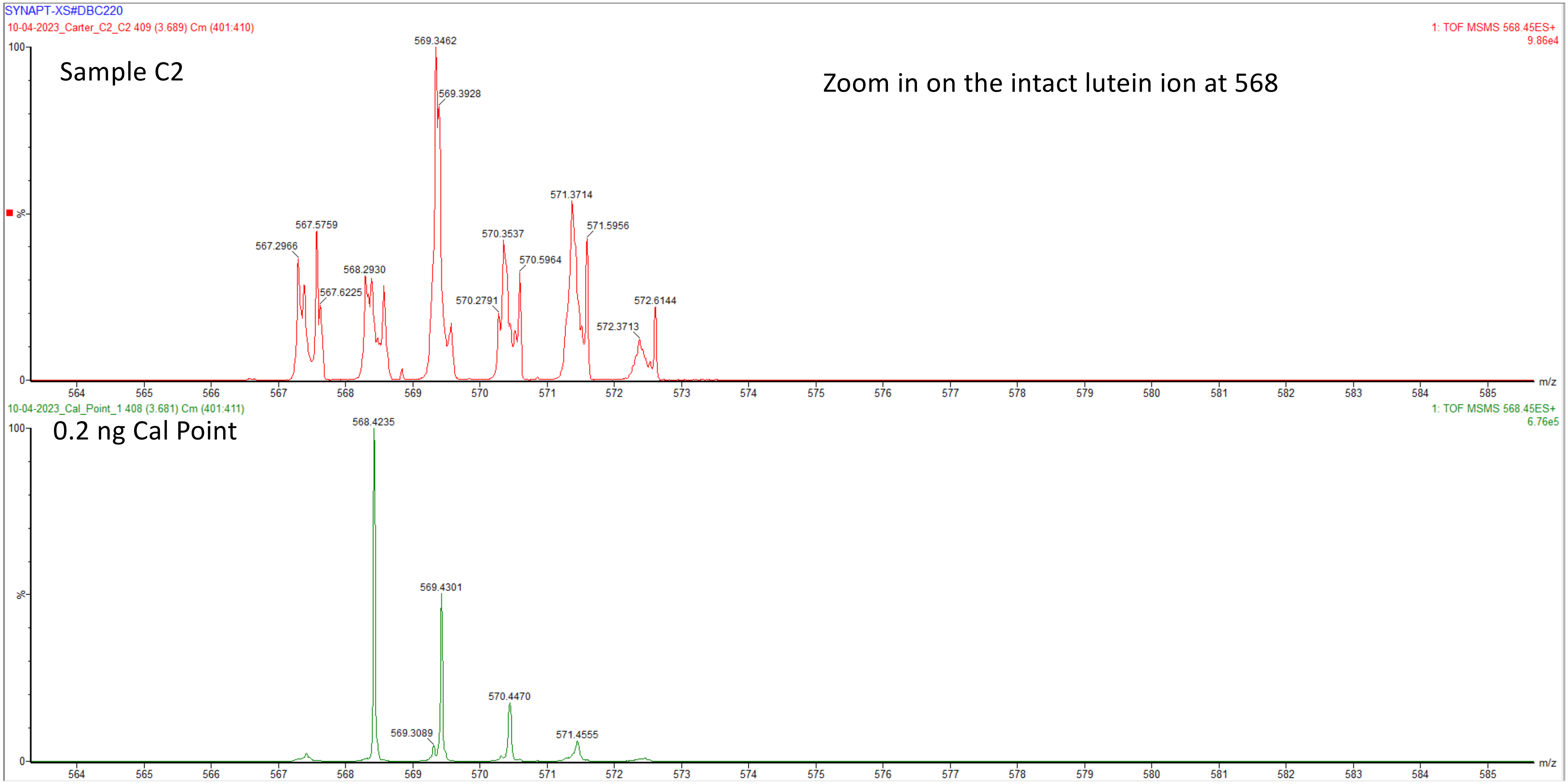

SYNAPT-XS#DBC220
10-04-2023_Carter_C2_C2 409 (3.689) Cm (401:410)
1: TOF MSMS 568.45ES+
9.86e4
Sample C2
Zoom in on the intact lutein ion at 568
569.3462
569.3928
571.3714
567.5759
571.5956
567.2966
570.3537
570.5964
568.2930
567.6225
570.2791
572.6144
572.3713
100
%
0
564
565
566
567
568
569
570
571
572
573
574
575
576
577
578
579
580
581
582
583
584
585
m/z
10-04-2023_Cal_Point_1 408 (3.681) Cm (401:411)
1: TOF MSMS 568.45ES+
6.76e5
0.2 ng Cal Point
568.4235
569.4301
570.4470
569.3089
571.4555
